# Supplementary material for: Lack of SMARCB1 expression characterizes a subset of human and murine peripheral T-cell lymphomas
Source: Nat Commun. 2024 Oct 3;15:8571. doi: 10.1038/s41467-024-52826-0 (PMC11452211; doi:10.1038/s41467-024-52826-0)
Supplement: Supplementary file 1 — Supplementary Information [file 41467_2024_52826_MOESM1_ESM.pdf]

**Supplementary Material**

# Lack of SMARCB1 expression characterizes a subset of human and murine peripheral T-cell lymphomas

Fischer et al.

## Inventory

- Supplemental Methods
- Supplemental References
- Supplemental Figure 1-12
- Supplemental Tables 1-11

Separate File: Supplemental Data 1-14

## Supplemental Methods

### Expression analysis of SMARCB1 in T-PLL using Western Blot

Protein expression of SMARCB1 in T-PLL was determined by Western blot analyses using the primary anti-BAF47/SMARCB1 antibody (BD Biosciences, cat# 612110 and cat# ab16645) and detection was performed using Species-specific HRP conjugated secondary antibodies. SuperSignal West Femto maximum sensitivity enhanced chemiluminescent (ECL) substrate (Thermofisher scientific, USA) was used for the detection of peroxidase activity from HRP-conjugated antibodies.

### Expression analysis of SMARCB1 in MF using IHC

We retrieved 15 cases of mycosis fungoides (MF) examined at the Pathology Section of the University Hospital of Careggi, Florence, between 2011 and 2023. The MF cohort comprehended 6 females and 9 males with a mean age of 57.7 and median age of 65 at the time of the diagnosis (range: 22-87 yrs) (**Table S2**). The cases included MF in early patch stage (n = 5; 33,3%), MF in plaque stage (n = 6; 40%), and MF in tumor stage (n = 4; 26,6%) of which 2 showed histological transformation (n = 2; 13,3%). Hematoxylin-eosin staining and immunohistochemical staining for lymphocytic (CD3, CD4, CD8, CD30) antigens were performed. Consequently, all cases were evaluated by immunohistochemistry for SMARCB1/INI-1 on 4-micron sections with the INI-1 mouse monoclonal antibody (MRQ-27 clone; Cell Marque) in a Ventana® XT instrument (Ventana Medical Systems, Inc. Tucson, AZ) according to manufacturer's instructions (Ventana).

### DNA sequencing and copy number analysis of the *SMARCB1* gene

DNA from tumor tissues was extracted using the GeneRead DNA FFPE Kit (Qiagen, Hilden, Germany). For targeted next-generation sequencing of SMARCB1 exons and flanking intronic sequences, the TruSight DNA target enrichment was used for library preparation and sequencing was performed on the MiSeq platform (Illumina Inc., San Diego, CA, USA). The generated fastq files were analyzed by SeqPilot software version 5.1.0 (Module SeqNext, JSI Medical Systems) for alignment and variant calling (hg19). For copy number determination, DNA was hybridized to an OncoScan CNV assay (Thermo Fisher Scientific, Waltham, MA, USA). Analysis was performed using the Chromosome Analysis Suite Software version 4.0 (Thermo Fisher Scientific, Waltham, MA, USA). Only copy number alterations larger than 50kb, encompassing at least 20 informative probes with a median log2ratio of >0.2 or <-0.2 and copy number neutral losses of heterozygosity larger than 5 Mb were considered for further

analyses. Additionally, FISH for the SMARCB1 locus was performed as described previously[1].

### HTG Transcriptome analysis

HTG Transcriptome Panel assay covers the vast majority of human mRNA transcripts with 19,616 probes (HTG Molecular Diagnostics, Inc., Tuscon, Arizona, USA). The assay was performed on FFPE-tissue sections (n = 9). After target protection, 4 µL were taken from each sample for library preparation with the HTG EdgeSeq (Illumina) Tag Pack (HTG Molecular Diagnostics, Inc., Tuscon, Arizona, USA) and the OneTaq® Hot Start 2X Master Mix in GC Buffer (NEB, Ipswich, Massachusetts, USA). After library purification with AMPure XP magnetic beads (Beckman Coulter Life Sciences, Brea, California, USA) according to HTG instructions, the purified libraries were quantified using the KAPA Library Quant Kit (Illumina) Universal qPCR mix (Roche, Basel, Switzerland) and the LightCycler 480 II (Roche, Basel, Switzerland). The libraries were subsequently sequenced using the Illumina NextSeq 500/550 High output v2.5 Reagent Kit (75 cycles) (Illumina, San Diego, California, USA). Quality control was performed using the HTG EdgeSeq Reveal Software (HTG Molecular Diagnostics, Inc., Tuscon, Arizona, USA). Only samples passing the QC criteria were considered in downstream analysis (n = 7). CPM values were plotted to show gene expression for SMARCB1.

### DNA methylation analysis using Illumina Infinium arrays

To measure DNA methylation 0.5-1 µg genomic DNA was bisulfite-converted using the EZ DNA Methylation kit (ZymoResearch, Irvine, CA, USA) according to the manufacturer's instructions. Due to the FFPE tissue, quality of the DNA was below average. Raw idat files generated from the human samples were normalized using the preprocess Illumina function without background correction from the minfi package[2] within the R statistical program (www.R-project.org, version 4.1.2). We converted human samples run on EPIC arrays into virtual 450K arrays in order to cross-compare all studies. For the mouse samples the raw idat files were normalized with GenomeStudio (v2011.1; methylation module 1.9.0; Illumina Inc., San Diego, CA, USA) applying default settings and internal normalization controls. Subsequently, beta values were calculated representing the percentage of DNA methylation at a certain cytosine base. For downstream analysis, loci with a detection p value > 0.01, rs loci and loci on gonosomes were excluded from further analysis. Differentially methylated loci for human and mouse samples were identified using the OMICS Explorer 3.6 (Qlucore; Lund, Sweden).

In order to identify meaningful hyper- or hypomethylated biological processes or molecular

functions a gene ontology enrichment analysis was performed using WEB-based Gene Set Analysis Toolkit[3]. Heatmaps, UMAPs (Uniform Manifold Approximation and Projection) and boxplots were generated in R using the pheatmap, umap and ggplot2 package, respectively.

**Cell lines**

*Smarcb1*-negative T15 cells (gift from Charles W. M. Roberts, Dana-Farber Cancer Institute, BOS, USA) and lymphoma cell lines (gift from Prof. Claudia Rössig, University Children's Hospital Münster, Münster, Germany;) Jurkat (T-ALL), Karpas-299, SR-786, SU-DHL-1 (all ALCL), Raji, Daudi (both Burkitt’s lymphoma) and U-937 (histiocytic lymphoma) were cultured as described in Supplementary Table 11 and maintained at 37°C and 5% $CO_2$.

Prior to all experiments, authentication of all cell lines was performed through STR profile PCR through the Institute for Forensic Medicine (University of Münster). All cell lines were regularly tested to be mycoplasma-free by PCR-analysis.

**Cell viability, apoptosis and cell cycle analysis**

For cell viability analysis, cells (4 x $10^3$ cell / 50 µl) were seeded in 96-well plates. Drugs were added twice, 24 h and 72 h after seeding, in ascending concentrations: HDACi SAHA was tested at 1 x $10^{-4}$, 1 x $10^{-3}$, 1 x $10^{-2}$, 1 x $10^{-1}$, 1, 10 and 100 µM. The inhibitor was obtained from Cayman chemical (#10009929). Three independent replicates with n = 4 technical replicates were analyzed. After 5 days of treatment, cell viability was measured via MTT assay using 10 µl MTT reagent (5 mg / ml MTT dissolved in PBS; Merck) and 4 h incubation time. Resulting formazan crystals were dissolved in 100 ml lysis buffer (isopropanol and 0,04 N HCl; purchased from SAV Liquid Production and Fisher Scientific) and the OD was evaluated spectrophotometrically at 570 nm and at the reference length of 630 nm by a Multiscan Ascent microplate reader (Thermo Fisher Scientific). $IC_{50}$ value and statistical analysis was calculated with GraphPad Prism 7 (GraphPad Software, La Jolla, CA, USA).

For apoptosis and cell cycle analysis of *Smarcb1*-negative PTCL-NOS cells, cells were seeded in 12-well plates (6.5 x $10^4$ cells / 650 µl) and treated 24 h and 72 h later. Drug concentrations were chosen based on the calculated $IC_{50}$ value: SAHA (0.25, 0.5, 1, 5 µM). All experiments were performed in biological triplicates with n = 3 technical replicates. For apoptosis detection, a FITC-Annexin V Apoptosis Detection Kit was utilized (BD Biosciences, #556547) following the manufacturer’s guidelines. After 5 days, cells and their supernatant were harvested and pelleted. 2 x $10^5$ cells were stained with Annexin-V and propidium iodide (PI) and incubated at room temperature for 15 min in the dark. Apoptotic Annexin-V positive

and necrotic PI positive cells we detected via flow cytometry. For cell cycle analysis 2 x $10^5$ cells were stained with DAPI solution (4', 6'-diamidino-2-phenylindole, AppliChem, #A4099) and incubated at room temperature for 15 min. Detection was performed using FACS (FACSCelesta from BD Biosciences). Data were analyzed with FlowJo software (version 10.1r1, Tree Star Inc., Ashland, OR, USA). For cell cycle phase analysis, the *Watson pragmatic algorithm* provided by the FlowJo software was applied. Statistical analysis (one-way ANOVA) was performed with Graphpad Prism 7.0 software (GraphPad Software, La Jolla, CA, USA).

### RNA isolation and bulk sequencing of murine T15 cell line

T15 cells were seeded in 6-well plates (2 x $10^6$ cell / 2 ml) and incubated with 1 µM SAHA for 72 h. In parallel, T15 cells lentivirally transduced with the *Smarcb1*-pInd20 vector (Addgene)[4], were induced with Doxycycline (0.5 µg/µl) for 72 h. Control T15 cells treated with vehicle for comparable time. RNA was isolated using RNeasy Mini Kit (QIAGEN, #74104) from cell pellets according to manufacturer's protocol. The experiment was performed in n = 3 technical replicates. RNA quality, purity and concentrations were determined using Bioanalyzer (Software Agilent 2100; Agilent Technologies, Inc). The cDNA libraries were run on the Illumina NextSeq 500 platform using the High Output 75 cycles kit at the Core Facility Genomics (CFG) of the Medical Faculty Münster. RNA sequencing was carried out by the CFG of the University Hospital Muenster, Germany, using the ultra II RNA directional library prep kit for Illumina (New England Biolabs, #E7760S) and the Next-Seq 500 sequencing platform (settings: high-output Kit, 75 Cycles v2.5 Chemie, 22 Mio single reads/sample). Raw FASTQ files were obtained and, after explorative quality control with FastQC[5] and MultiQC[6], Salmon[7] was used for pseudo-alignment and quantification of the samples to the mouse transcriptome (downloaded from Ensembl, release 94). Default parameters were used. Further analyses were performed in R. We employed the Bioconductor package tximport[8] to summarize transcript-level estimates computed by Salmon for a gene-level analysis. To find differentially expressed genes, we used the package DESeq2[9] and tested for SMARCB1 re-expression *versus* control conditions. Only genes with adjusted p-value $< 0.05$ were considered (Benjamini-Hochberg correction).

### Histological and multiplex immunofluorescence analysis of murine spleen samples

Protocols and animal housing were in accordance with all guidelines provided by the local regulatory authorities (reference number TVA-84-02.04.2018.A296; Government of NRW,

Germany). For histology, isolated murine spleens were fixed in 4% paraformaldehyde overnight, immersed in 30% sucrose for two days, followed by tissue embedding in O.C.T.™ Compound (Tissue-Tek Sakura) and stored at -80 °C. Tissues were cryo-sectioned into 12 µm slices. For multiplexed immunofluorescence analysis, slices of PTCL-NOS$^{Smarcb1-}$ and corresponding murine control spleens were stained in the MACSima imaging system (Miltenyi Biotec) using antibodies against B220 (RA3-6B2, Miltenyi Biotec, APC, 1:50), Ly6G (1A8, Miltenyi Biotec, PE, 1:50) and EZH2 (REA907, Miltenyi Biotec, APC, 1:50). Nuclei were counterstained with DAPI. Micrograph images were aquired with the build-in 20× long working distance objective (Numerical Aperture 0.45) and sCMOS camera with a pixel size of 0.17 µm x 0.17 µm.The acquired pictures were stitched and preprocessed using MACS iQ View Analysis Software (Miltenyi Biotec, Germany), and representative overlay pictures were generated for Figure 5C. No processing or averaging was performed which enhances the resolution of the image. All post-processing adjustments were applied to the entire image and no non-linear adjustments/gamma changes were made; however, we did adjust individual color channels in the images shown in Figure 5C using Photoshop version 25.9.1 (Adobe) to increase image contrast for the print version. To quantify the numbers of tumor cells, B-cells and neutrophils, representative areas/ROIs (regions of interest) of 1500 x 1500 µm were analyzed. For this purpose, cells were segmented based on the DAPI signal using the StarDist plugin[10] in ImageJ (NIH, USA) and MACS iQ View and up to 65,536 cells analyzed per ROI. Tumor cells were then defined as EZH2$^{high}$, B-cells as B220$^{+}$ and neutrophils as Ly6G$^{+}$ cells using threshold values for each marker.

**Sample processing for scRNA-seq of murine Smarcb1-negative PTCL samples**

For single-cell preparation, murine spleens were collected independently of gender and minced using scalpels. Enzymatic (StemPro™ Accutase™ (Gibco™, #A1110501); 37°C) and mechanical dissociation were applied for 20 min. Afterwards, cells were washed with PBS. Erythrocytes were lysed using ACK lysing buffer (Gibco™, #A1049201) according to the manufacturer’s protocol. Then, to remove non-viable cells, they were stained with 7-AAD (eBioscience™, #00-6993-50) and sorted (BD FACSAria-II). Manual cell counting of sorted cells was performed using Trypan blue staining. The single-cell suspension was processed further using Chromium Single Cell 3’ Gel Bead Kit v2 (10x Genomics) according to the manufacturer’s protocol. In short, single-cell GEMs (Gel Beads in Emulsion) were generated on the Chromium Controller, followed by GEM-RT, Dyna Beads cleanup, cDNA amplification and SPRIselect beads cleanup. The Library Bead Kit and Chromium i7 Multiplex Kit was used for generating indexed single-cell libraries for Illumina sequencing. Quality, purity, size and concentrations of cDNA and libraries were determined by Tapestation 2000 (Agilent

Technologies, Inc). Libraries were sequenced using the Next-Seq 500 sequencing platform (high-output Kit, 75 Cycles v2 Chemie) at the Genomics Core Facility (University Hospital Münster, Münster). Sequencing was performed using the Next-Seq 500 sequencing platform. Raw data were processed by CellRanger (10X Genomics) and then analyzed using custom R and python scripts.

**Sample processing for scRNA-seq of human SMARCB1-negative PTCL samples**

For single-cell RNA sequencing of human SMARCB1-negative tumors, we used 10X Genomics FLEX technology (16x format) and essentially followed the protocols provided by the manufacturer. Briefly, cell isolation from archival FFPE material was performed according to protocol CG000632_RevB. After deparaffinization and rehydration, tissue sections were dissociated using a heated gentleMACS Octo-Dissociator for FFPE tissue (Miltenyi Biotec) with a freshly prepared Dissociation Enzyme Mix containing Liberase TH (Millipore Sigma). After sample filtration, single-nucleus suspensions were counted using the Invitrogen Countess 3 Automated Cell Counter (Thermo Fisher Scientific). To generate fixed RNA gene expression libraries, we used the Chromium Next GEM Single Cell Fixed RNA Sample Preparation Kit, 16 rxn (PN-1000414). Single-nucleus suspensions were mixed with human transcriptome probes and hybridized for 20 hours at 42°C and then processed according to protocol CG000527_RevE for multiplex libraries. One million cells per sample were used for probe hybridization. GEMs were prepared with a targeted recovery of 80,000 cells in each 16-plex library.

**Bioinformatics analysis of scRNA-seq data**

Note: * indicates settings/specifications for mouse scRNA-seq samples; ** for human Fixed RNA samples.

Briefly, we used the R package Seurat[11] to perform initial quality control and filtering, integration, dimensionality reduction, clustering and differentially expressed gene (DEG) analysis. The raw Illumina bcl files were demultiplexed using Cell Ranger (*v.3.0.2 and **v.7.1.0) 'mkfastq' step with default specifications. Individual sample gene expression matrices were generated using the Cell Ranger *count and Cell Ranger **multi pipeline using genome version *mm10 and **GRCh38-2020-A provided by 10X Genomics Cell Ranger. Data analysis was performed using R (*v.3.6.1 and **v.4.3.1) and Seurat[11] (both v.3.1.3[12] and v.4.3.0[13]).

For initial quality control by Seurat, genes that were expressed in fewer than three cells and cells that expressed fewer than *50 genes and **200 genes were excluded from analysis. Briefly, each dataset was filtered on dataset-specific parameters for genes per cell, UMIs per cell and percentage of mitochondrial genes. We performed normalization using the Seurat function *NormalizeData* (method = "LogNormalize", scale.factor = 10,000). Finally, highly variable genes (n = 2,000) were calculated with the selection method "vst".

To integrate samples from different conditions, we applied Seurat *v3 integration and Seurat **v4 integration method. Each sample was considered as one batch. For human sample integration, reciprocal PCA (RPCA) method from Seurat was applied. This procedure is meant to correct for possible batch effects present in the data, while maintaining biological variation. After this step, we performed scaling, PCA, dimensionality reduction (using UMAPs) and clustering (using Louvain algorithm) on the integrated dataset. Clustering resolution was initially set arbitrarily at 0.5. However, for the mouse dataset, we sub-clustered 2 clusters that we found being composed of different populations by analyzing the expression of marker genes. Differential gene expression was computed using MAST algorithm[14] (murine samples) or the FindMarkers function of Seurat (human samples). Statistically significant genes were considered having a q-value <0.05 (Bonferroni correction). For murine samples, we considered at first only PTCL and control samples, extending to the PTCL-SAHA samples only at a second step. Cell type annotation was performed as described below. To identify tumor cells in mouse PTCL, we used a gene expression signature of *Ezh2*, *Uhrf1*, *Tox2*, *Pdcd1* and *Smarcb1*. In particular, we classified tumor cells by having a normalized gene expression of *Ezh2*, *Uhrf1*, *Tox2*, *Pdcd1* > 2 (OR) and *Smarcb1* = 0.

For subsetting of Myeloid and Tumor/T-cell compartments in human single-cell data, all clusters containing tumor cells and T-cells (Tumor/T-cell subset) or monocytes/macrophages (Myeloid subset) were isolated from the integrated human Fixed RNA object and the entire Seurat pipeline run again. We selected the clusters identified using resolution 0.4 for downstream analysis.

**Reanalysis of publicly available datasets (murine control spleens)**

Published scRNA-seq data of mouse healthy spleen was retrieved from the Tabula Muris Consortium[15,16]. Related information can be found under:

https://figshare.com/articles/dataset/Single-cell_RNA-seq_data_from_microfluidic_emulsion_v2_/5968960.

In particular, we used raw count matrices that were processed by the 10X Genomics Platform and selected only samples derived from the spleen tissue (two samples: “Spleen-10X_P4_7" and “Spleen-10X_P7_6”) for downstream analysis.

### Cell type annotation

Cell type annotation of the scRNA-seq data sets of human and mouse PTCL was performed through an interplay of bioinformatics analyses and manual curation. First, cluster-specific DEG (differentially expressed gene) lists were created for the respective Seurat objects (see **Supplementary Data S3, 9, 14**). For further preselection of suitable marker genes, these lists were then filtered according to (i) upregulated genes, (ii) the average fold change (avg_log2FC) of these upregulated genes and (iii) their "delta_pct", i.e. the difference of pct. 1 (= percentage of cells expressing this gene in the examined cluster) and pct.2 (= percentage of expressing cells in all remaining clusters), which provides a measure of the “signal-to-noise” ratio of the relative expression strength of a gene. In cases of uncertainty or ambiguity, this process was repeated or refined by subclustering of individual clusters or subsetting of cognate meta-clusters (e.g. the Tumor/T-cell and Myeloid subsets from human PTCL) and subsequent re-clustering. The genes selected in this way (high values for avg_log2FC and delta_pct) were then manually reviewed and curated. This was done through comparisons with established cell type-specific marker gene lists, publicly available reference data sets including various scRNA-seq cell atlases and through targeted queries of public domain databases (e.g. NCBI Pubmed, NCBI Gene, STRING), public domain tools (e.g. ToppGene Suite) and various search engines for classifying genes with unknown functions and/or cell type specificity.

### GSEA analysis

Gene set enrichment analysis (GSEA) for human tumor cluster T9 and myeloid cluster M0 was performed using clusterProfiler[17] (v.4.8.2 R package) and run using MSigDB[18] gene sets C2: CP REACTOME.  Enrichment results were filtered to a Benjamini–Hochberg adjusted P-value $< 0.05$.

## Functional annotation of clusters using published cancer hallmark metaprograms

For the functional annotation of clusters from the Tumor/T-cell and Myeloid subsets of human PTCL and clusters of the murine WT/PTCL samples, a comparison with published cancer hallmark metaprograms MP1 to MP41[19] was carried out. The latter each contain 50 signature genes. These were compared with the upregulated (based on avg_log2FC) DEGs of the individual clusters of the Tumor/T-cell subset (T0-T12), of the Myeloid subset (M0-M6) and of the WT/PTCL dataset (C0-C23). The number of hits then served as an indication of the presence of a particular metaprogram in the individual clusters. For visualization, these results were displayed in the form of a heatmap using the freely available web server Heatmapper (http://heatmapper.ca/).

## Analysis of ligand-receptor interaction-based cell-cell communication

To predict the occurrence of cell-cell communication based on ligand-receptor (L-R) interactions in our data, we used the Python package available with *CellPhoneDB[20] (*v2 for mouse data, ** for human data), a publicly available repository of L-R interactions. Since the interactions are only annotated for human, we considered a subset of homologous genes between human and mouse for the analysis of murine data, using the R package homologene (https://CRAN.R-project.org/package=homologene). We run the statistical analysis implemented in CellPhoneDB with default parameters. For downstream analysis, we used the R/Shiny application InterCellar[21].

## Pseudotime trajectories of murine scRNA-seq data

To infer trajectories of the lymphoid cell population of murine PTCL and SAHA scRNA-seq samples, we used the python package STREAM[22]. The beginning of the pseudotime was empirically determined by choosing the branch with the highest enrichment of cells belonging to cluster 1, which showed a phenotype typical of naive T-cells.

## Supplementary References


1. Fruhwald, M.C. *et al.* Non-linkage of familial rhabdoid tumors to SMARCB1 implies a second locus for the rhabdoid tumor predisposition syndrome. *Pediatr Blood Cancer* **47**, 273-8 (2006).
2. Aryee, M.J. *et al.* Minfi: a flexible and comprehensive Bioconductor package for the analysis of Infinium DNA methylation microarrays. *Bioinformatics* **30**, 1363-9 (2014).
3. Zhang, B., Kirov, S. & Snoddy, J. WebGestalt: an integrated system for exploring gene sets in various biological contexts. *Nucleic Acids Res* **33**, W741-8 (2005).
4. Meerbrey, K.L. *et al.* The pINDUCER lentiviral toolkit for inducible RNA interference in vitro and in vivo. *Proc Natl Acad Sci U S A* **108**, 3665-70 (2011).
5. Andrews, S. FastQC: A Quality Control Tool for High Throughput Sequence Data *[Online]* (2010).
6. Ewels, P., Magnusson, M., Lundin, S. & Kaller, M. MultiQC: summarize analysis results for multiple tools and samples in a single report. *Bioinformatics* **32**, 3047-8 (2016).
7. Patro, R., Duggal, G., Love, M.I., Irizarry, R.A. & Kingsford, C. Salmon provides fast and bias-aware quantification of transcript expression. *Nat Methods* **14**, 417-419 (2017).
8. Soneson, C., Matthes, K.L., Nowicka, M., Law, C.W. & Robinson, M.D. Isoform prefiltering improves performance of count-based methods for analysis of differential transcript usage. *Genome Biol* **17**, 12 (2016).
9. Love, M.I., Huber, W. & Anders, S. Moderated estimation of fold change and dispersion for RNA-seq data with DESeq2. *Genome Biol* **15**, 550 (2014).
10. Schmidt, U., Weigert, M., Broaddus, C., Myers, G. Cell Detection with Star-Convex Polygons. *Frangi, A., Schnabel, J., Davatzikos, C., Alberola-López, C., Fichtinger, G. (eds) Medical Image Computing and Computer Assisted Intervention – MICCAI 2018. Springer, Cham* **vol 11071**(2018).
11. Butler, A., Hoffman, P., Smibert, P., Papalexi, E. & Satija, R. Integrating single-cell transcriptomic data across different conditions, technologies, and species. *Nat Biotechnol* **36**, 411-420 (2018).
12. Stuart, T. *et al.* Comprehensive Integration of Single-Cell Data. *Cell* **177**, 1888-1902.e21 (2019).
13. Hao, Y. *et al.* Integrated analysis of multimodal single-cell data. *Cell* **184**, 3573-3587.e29 (2021).
14. Finak, G. *et al.* MAST: a flexible statistical framework for assessing transcriptional changes and characterizing heterogeneity in single-cell RNA sequencing data. *Genome Biol* **16**, 278 (2015).
15. Tabula Muris, C. A single-cell transcriptomic atlas characterizes ageing tissues in the mouse. *Nature* **583**, 590-595 (2020).
16. Tabula Muris, C. *et al.* Single-cell transcriptomics of 20 mouse organs creates a Tabula Muris. *Nature* **562**, 367-372 (2018).
17. Wu, T. *et al.* clusterProfiler 4.0: A universal enrichment tool for interpreting omics data. *Innovation (Camb)* **2**, 100141 (2021).
18. Subramanian, A. *et al.* Gene set enrichment analysis: a knowledge-based approach for interpreting genome-wide expression profiles. *Proc Natl Acad Sci U S A* **102**, 15545-50 (2005).
19. Gavish, A. *et al.* Hallmarks of transcriptional intratumour heterogeneity across a thousand tumours. *Nature* **618**, 598-606 (2023).
20. Efremova, M., Vento-Tormo, M., Teichmann, S.A. & Vento-Tormo, R. CellPhoneDB: inferring cell-cell communication from combined expression of multi-subunit ligand-receptor complexes. *Nat Protoc* **15**, 1484-1506 (2020).
21. Interlandi, M., Kerl, K. & Dugas, M. Interactive analysis and exploration of cell-cell communication in single-cell transcriptomics with InterCellar. *Research Square*

https://doi.org/10.21203/rs.3.rs-525466/v1(2021).
22. Chen, H. *et al.* Single-cell trajectories reconstruction, exploration and mapping of omics data with STREAM. *Nat Commun* **10**, 1903 (2019).
23. Szklarczyk, D. *et al.* The STRING database in 2023: protein-protein association networks and functional enrichment analyses for any sequenced genome of interest. *Nucleic Acids Res* **51**, D638-d646 (2023).

## Figure S1

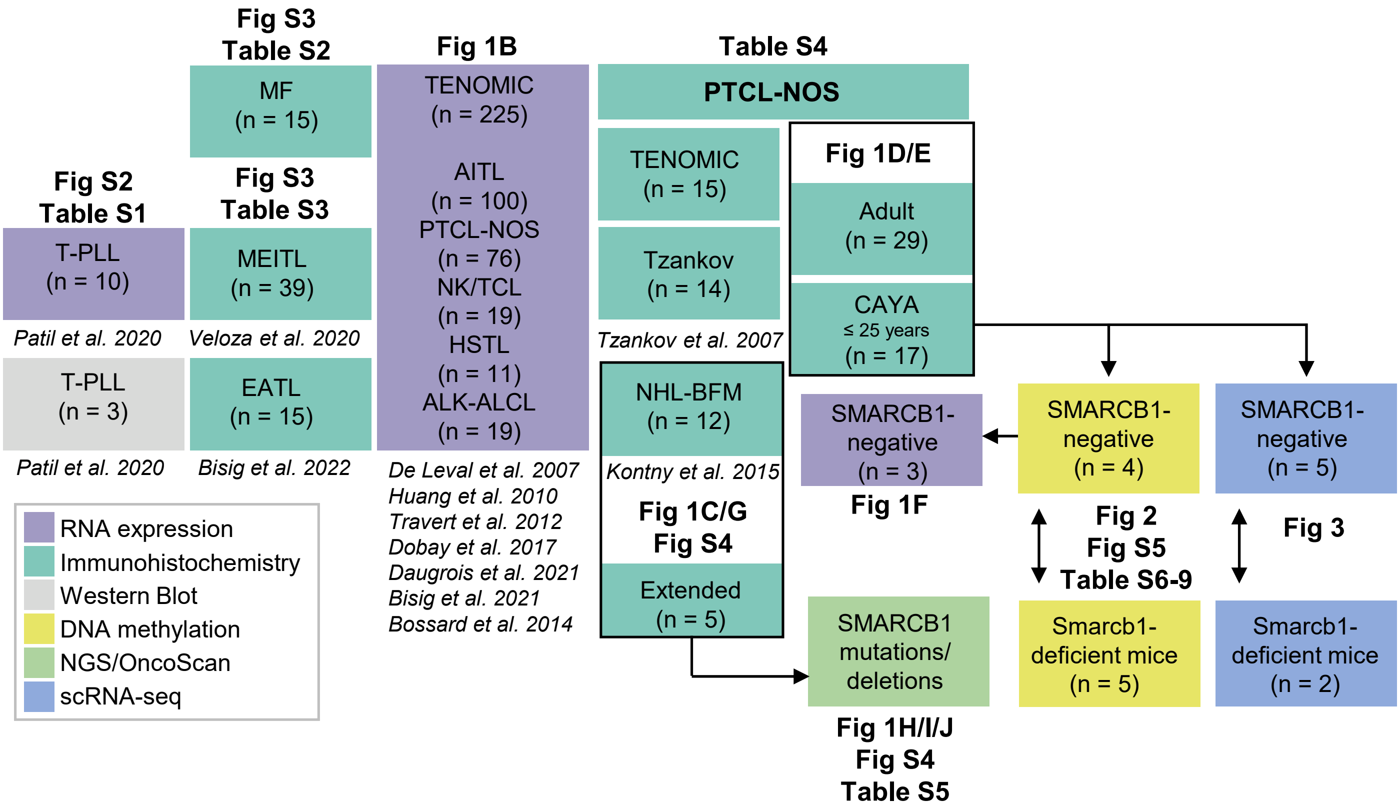


**Suppl. Figure 1. Overview of the human cohorts analyzed in this study.** The study included RNA and protein expression data of T-cell prolymphocytic leukemia (T-PLL), immunohistochemistry of intestinal T-cell lymphomas (MEITL and EATL), RNA expression data of a large cohort of peripheral T-cell lymphomas including AITL, PTCL-NOS, NK/TCL, HSTL, ALK-negative ALCL. Additionally, immunohistochemistry was performed in selected PTCL-NOS cases. Of identified SMARCB1-negative PTCL-NOS cases SMARCB1 mutations and deletions and DNA methylation was analyzed and compared to the murine model.

T-PLL, T-cell prolymphocytic leukemia; MF, mycosis fungoides; MEITL, monomorphic epitheliotropic intestinal T cell lymphoma; EATL, enteropathy-associated T-cell lymphoma; PTCL-NOS, Peripheral T cell lymphoma not otherwise specified; AITL, Angioimmunoblastic T cell lymphoma; NKTCL, Natural killer/ T cell lymphoma; HSTL, Hepatosplenic T cell lymphoma; ALCL-ALK-, ALK-negative anaplastic large cell lymphoma; CAYA, children, adolescents, and young adults.

## Figure S2

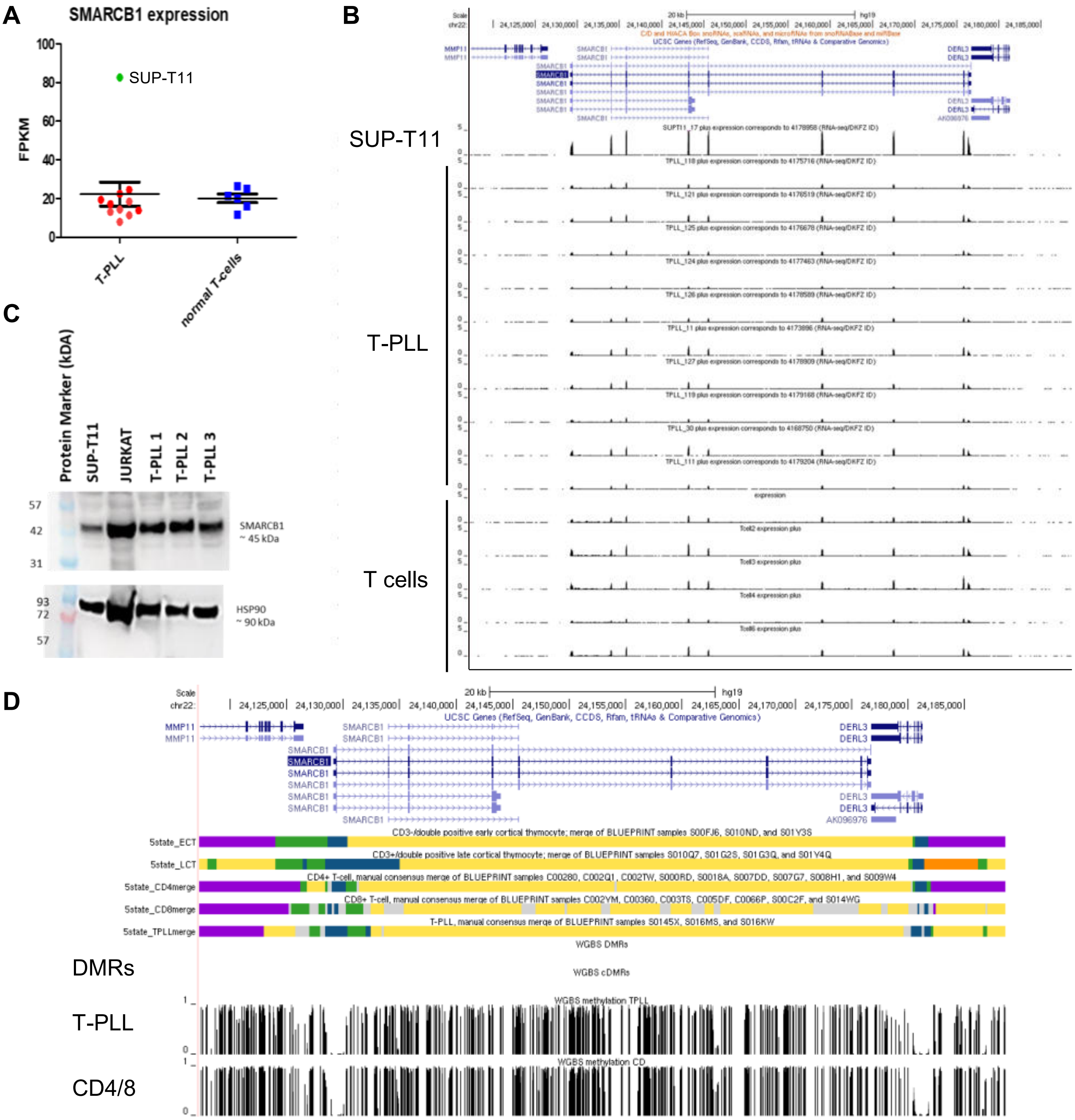


**Suppl. Figure 2. SMARCB1 expression analyses in T-PLL and healthy T-cells.** (A) Scattered boxplot showing the RNA expression values in FPKM (fragments per kilobase million) of SMARCB1. The vertical bars indicate the mean with SEM (standard error of the mean). (B) Within the UCSC genome browser (GENCODE version 19) track bars indicate SMARCB1 expression in 10 primary T-PLL cases with inv(14)/t(14;14) and SUP-T11 cell line in comparison to non-malignant CD4+ and CD8+ T-cells. Depicted is the strand-specific expression of the negative strand of the SMARCB1 locus. (C) Western blot of SMARCB1 in T-cell leukemia (SUP-T11 and JURKAT) and three T-PLL primary samples using anti-SMARCB1 antibody. (D) UCSC ref seq genes are displayed and below the chromatin states for the thymic and mature T-cells along with the single and merged chromatin states at the SMARCB1 locus for three T-PLL cases are shown. Below that DMRs detected in T-PLL by comparing three T-PLL cases and five normal CD4+ and CD8+ T-cells are shown. The next two tracks show the percentage of DNA methylation determined by WGBS at the SMARCB1 locus non-malignant CD4+ and CD8+ T-cells combined and T-PLL (three cases combined). Source data of panels A and C are provided as a Source Data file.

**Figure S3**

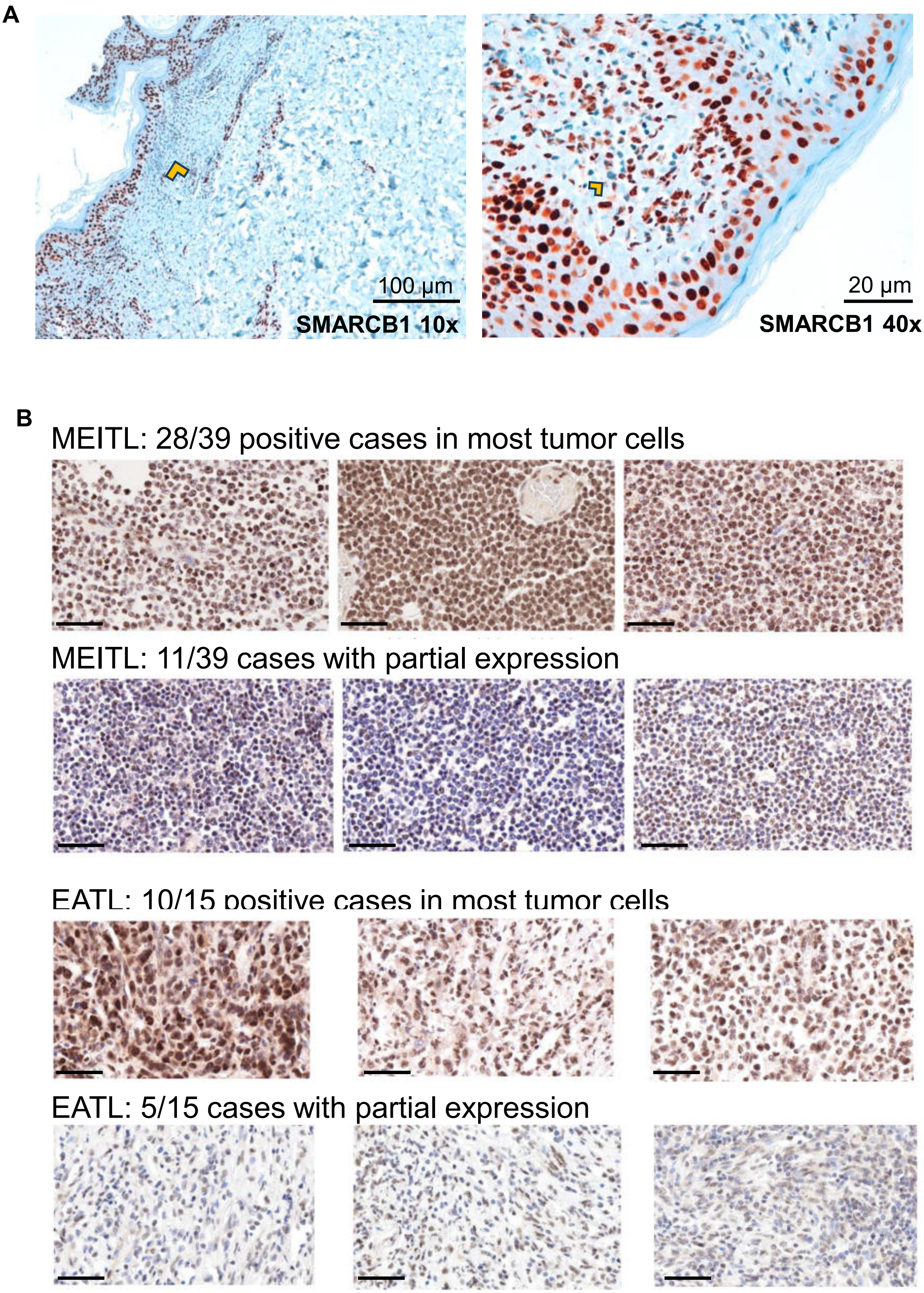


**Suppl. Figure 3: SMARCB1 (INI1) protein expression in MF, MEITL and EATL cases.** (A) SMARCB1 IHC staining of mycosis fungoides (MF) cases. Most of the MF cases (n = 15) showed indistinctively intense nuclear staining (compare **Table S2**). Only case number 2 displayed a few scattered negative elements with irregular nuclear profiles located in the superficial dermis (5%) and positive staining in most of the tumor cells (95%). Scale bar 100 µm left picture; 20 µm right picture. (B) SMARCB1 (INI1) protein expression was investigated by immunohistochemistry in 39 monomorphic epitheliotropic intestinal T cell lymphoma (MEITLs) and 15 enteropathy-associated T-cell lymphoma (EATLs). While most cases show positivity of SMARCB1 staining in the majority of tumor cells (90-100%), 28% of MEITLs and 33% of EATLs show a staining with partial loss of SMARCB1 (≤ 70% of IHC expression, **Table S3**). 40x magnification, Scale bar 50 µm.

**Figure S4**

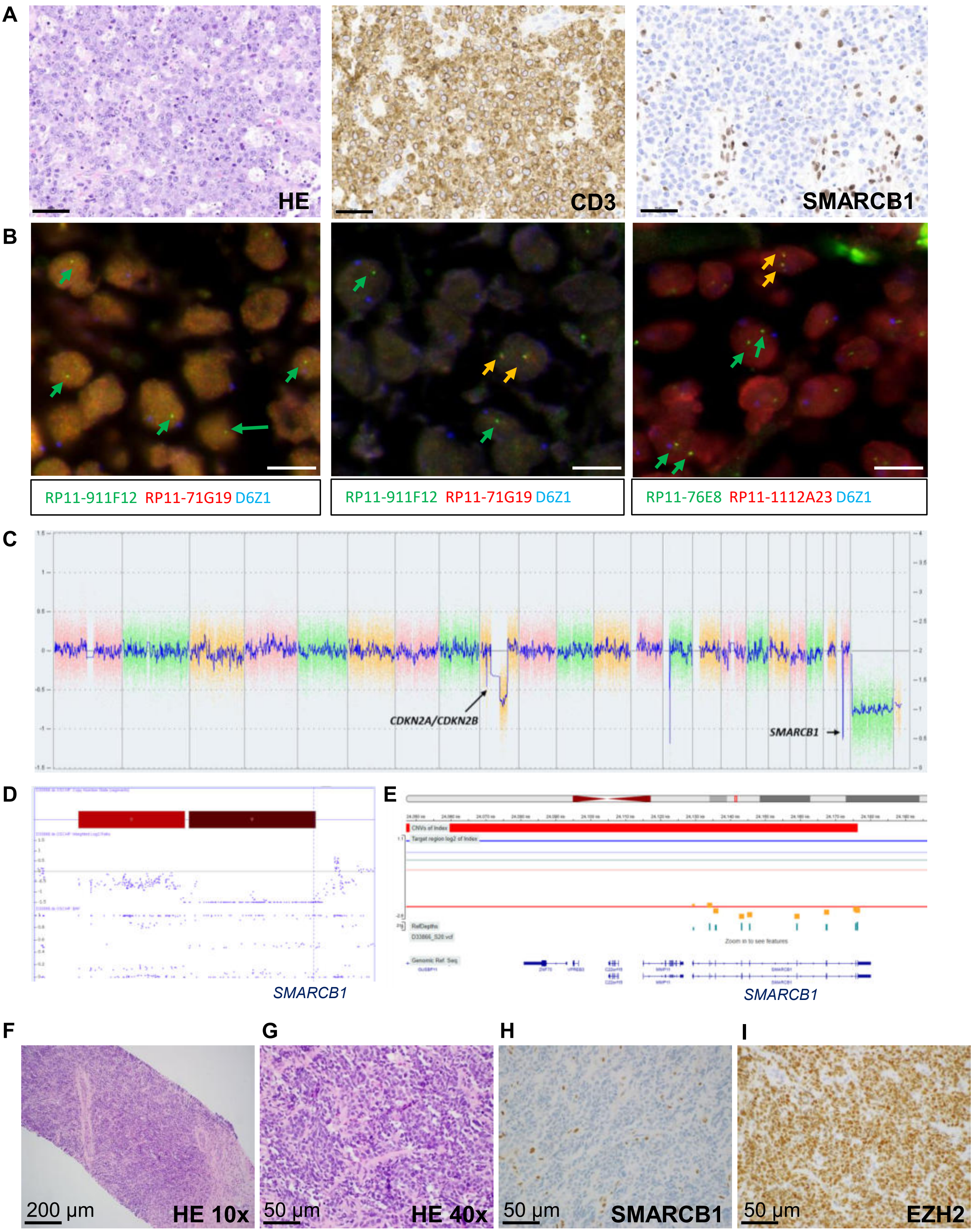

A
HE
CD3
SMARCB1
B
RP11-911F12 RP11-71G19 D6Z1
RP11-911F12 RP11-71G19 D6Z1
RP11-76E8 RP11-1112A23 D6Z1
C
CDKN2A/CDKN2B
SMARCB1
D
SMARCB1
E
SMARCB1
F
200 µm
HE 10x
G
50 µm
HE 40x
H
50 µm
SMARCB1
I
50 µm
EZH2

**Suppl. Figure 4: Detailed histopathological and genomic characterization of patient 4 and 8.** (A-E) Analysis of PTCL-NOS CAYA case (patient 5, age = 9). (A) Immunohistochemical analysis shows SMARCB1 negativity in CD3 positive tumor cells. HE, CD3 and SMARCB1 (INI1) staining are shown. Scale bar 50 µm. (B) Results from FISH analysis on paraffin embedded tumor sections using two different FISH assays for the SMARCB1 locus in 22q11 show a homozygous deletion of the BAC clones labelled in spectrum orange (RP11-71G19 and RP11-1112A23) and a heterozygous deletion for the BAC clone RP11-911F12 labelled in spectrum green. Green arrows: signals from BAC clones labelled in spectrum green in tumor cells. Yellow arrows: colocalized signals correspond to fusion signals indicating intact SMARCB1 loci in normal cells. Scale bar 5 µm. (C) Copy number profile obtained by OncoScan array analysis. Log2 ratios are shown in a whole genome view. Deletion of the SMARCB1 locus is indicated. (D) OncoScan array showing deletion on chromosome 22. The SMARCB1 locus is homozygously deleted. Log2 ratios and BAF are shown. (E) SMARCB1 sequencing confirmed homozygous deletion. Log2 ratios are shown. (F-I) SMARCB1 deficient PTCL case (patient 8, age = 24). Peripheral T cell lymphoma of the right ovary/adnexa with unclear maturation. The lymphoma was positive for CD3, CD2, CD8, CD99 and CD117 and negative for CD20, CD1a, CD5, CD7, CD4, CD10, CD30 and CD56 (not shown) as well as for SMARCB1 (H). Additionally, the lymphoma is positive for EZH2 (I). Scale bar 200 µm (F); 50 µm (G-I).

**Figure S5**

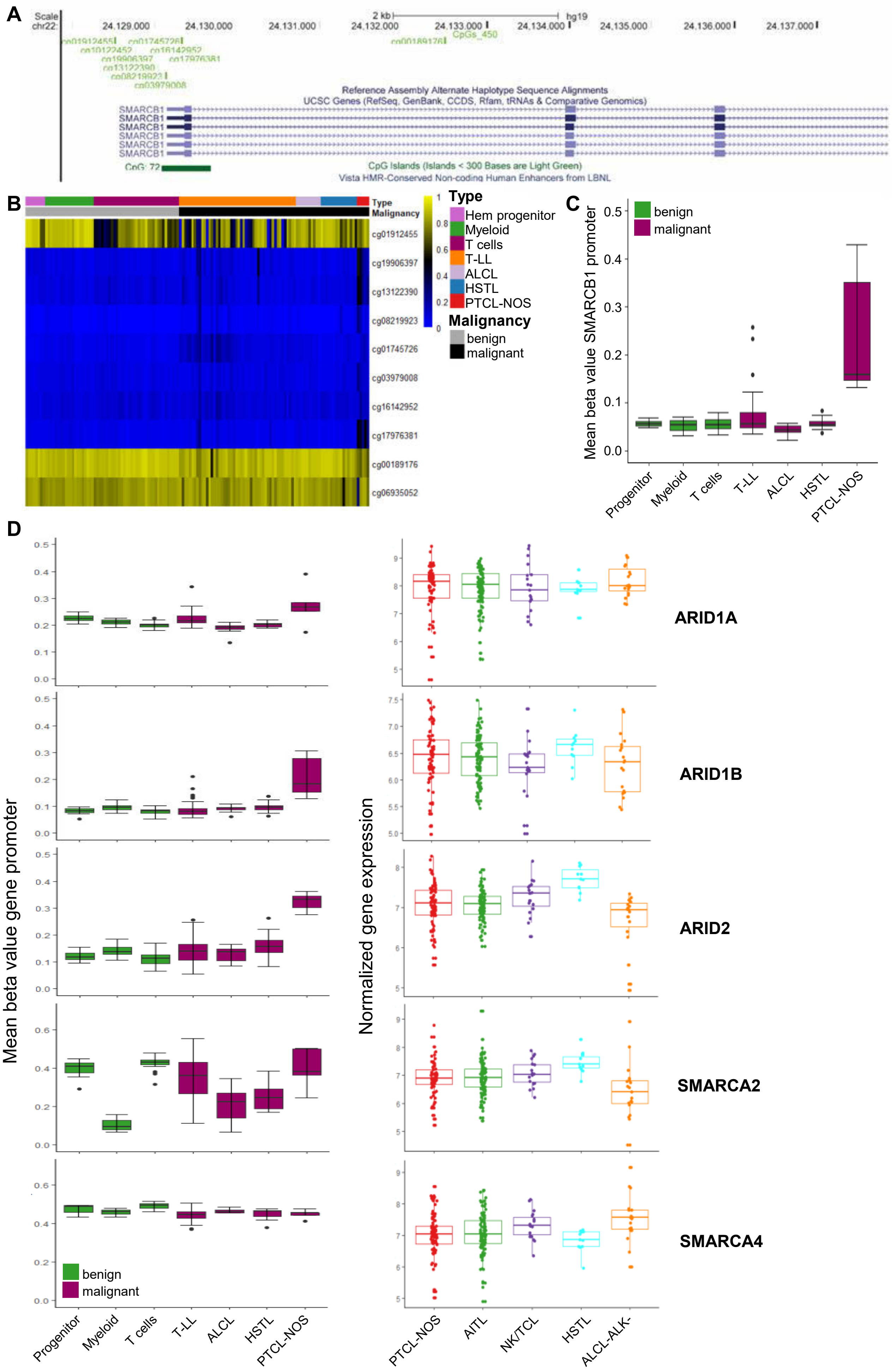

A
Scale
chr22:
2 kb
hg19
24.129.000
24.130.000
24.131.000
24.132.000
24.133.000
24.134.000
24.135.000
24.136.000
24.137.000
CpGs_450
Reference Assembly Alternate Haplotype Sequence Alignments
UCSC Genes (RefSeq, GenBank, CCDS, Rfam, tRNAs & Comparative Genomics)
SMARCB1
CpG: 72
CpG Islands (Islands < 300 Bases are Light Green)
Vista HMR-Conserved Non-coding Human Enhancers from LBNL
B
Type
Malignancy
cg01912455
cg19906397
cg13122390
cg08219923
cg01745726
cg03979008
cg16142952
cg17976381
cg00189176
cg06935052
1
0.8
0.6
0.4
0.2
0
Type
Hem progenitor
Myeloid
T cells
T-LL
ALCL
HSTL
PTCL-NOS
Malignancy
benign
malignant
C
benign
malignant
Mean beta value SMARCB1 promoter
0.5
0.4
0.3
0.2
0.1
0.0
Progenitor
Myeloid
T cells
T-LL
ALCL
HSTL
PTCL-NOS
D
Mean beta value gene promoter
Normalized gene expression
ARID1A
ARID1B
ARID2
SMARCA2
SMARCA4
benign
malignant
Progenitor
Myeloid
T cells
T-LL
ALCL
HSTL
PTCL-NOS
PTCL-NOS
AITL
NK/TCL
HSTL
ALCL-ALK-

**Suppl. Figure 5: DNA methylation and expression of SMARCB1 and other SWI/SNF members in benign and malignant T cells.** (A) Localization of array CpGs around the *SMARCB1* promoter. UCSC screenshot showing localization of CpGs from the Infinium HumanMethylation450 BeadChip in the area flanking the *SMARCB1* promoter. (B) Heatmap showing methylation at 10 CpGs at the *SMARCB1* locus. DNA methylation beta values are shown for 140 samples (listed in **Table S6**). The PTCL samples with the mutation/deletion (patient 1) is located at the left site of the PTCL group (red). (C) SMARCB1 promoter methylation in benign and neoplastic T cells. Mean is shown for three CpGs in the SMARCB1 promoter (cg19906397, cg13122390, cg08219923). Wilcoxon test was performed to compare PTCL and benign (p = 0.000897) and neoplastic (p = 0.00126) subtypes. The PTCL sample with the mutation/deletion (patient 1) shows the highest mean beta value (0.429). Boxplot settings: middle, median; lower hinge, 25% quantile; upper hinge, 75% quantile; upper/lower whisker, largest/smallest observation less/greater than or equal to upper/lower hinge ±1.5 * IQR. (D) Promoter DNA methylation and expression levels of SWI/SNF members in T cell lymphomas. Normalized expression is shown. Promoter methylation in benign and neoplastic T cells. Mean is shown for all CpGs in the promoter region of the gene. RNA expression in samples from the TENOMIC study (n = 225) for genes which are part of the SWI/SNF complex. PTCL-NOS, Peripheral T cell lymphoma not otherwise specified (n = 76); AITL, Angioimmunoblastic T cell lymphoma (n = 100); NKTCL, Natural killer/ T cell lymphoma (n = 19); HSTL, Hepatosplenic T cell lymphoma (n = 11); ALCL-ALK-, ALK-negative anaplastic large cell lymphoma (n = 19). Source data of panels C and D are provided as a Source Data file. Boxplot settings: middle, median; lower hinge, 25% quantile; upper hinge, 75% quantile; upper/lower whisker, largest/smallest observation less/greater than or equal to upper/lower hinge ±1.5 * IQR. Figure Created with BioRender.com released under a Creative Commons Attribution-NonCommercial-NoDerivs 4.0 International license.

# Supplementary Figure 6

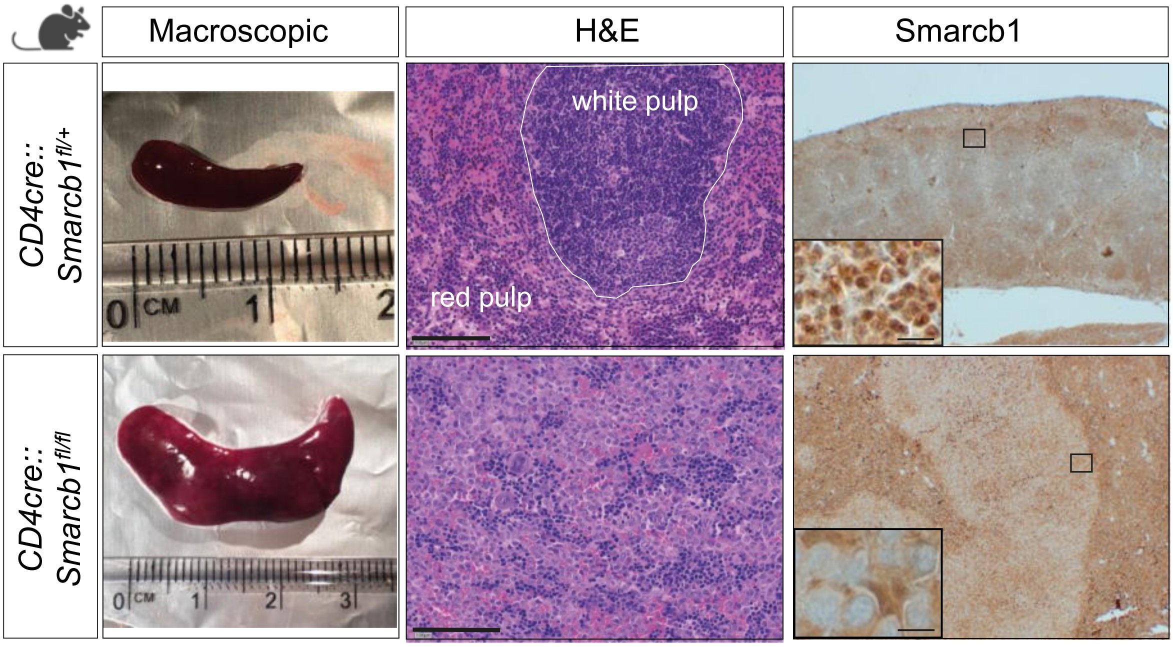


**Suppl. Figure 6. Histopathological characterization of lymphomas induced in Smarcb1-deficient mice.** CD4cre::Smarcb1$^{fl/fl}$ mice present splenomegaly (d) with alteration of tissue morphology (e, H&E) and loss of Smarcb1 positivity compared to control mice CD4cre:: Smarcb1$^{fl/+}$. Images were captured with an Olympus BX43 microscope (Olympus K. K., Tokio, Japan) equipped with 2x/0.08, 4x/0.10, 10x/0.25 and 20x/0.40 objectives and an Olympus SC50 camera. Images were processed with Adobe Illustrator Software 25.4.1 (Adobe Systems, San Jose, CA). Scale bars: 100 µm (b, e); 10 µm (c´, f´). Original magnification 2x (c, f), 20x (b, e), 40x (c´, f´).

# Supplementary Figure 7

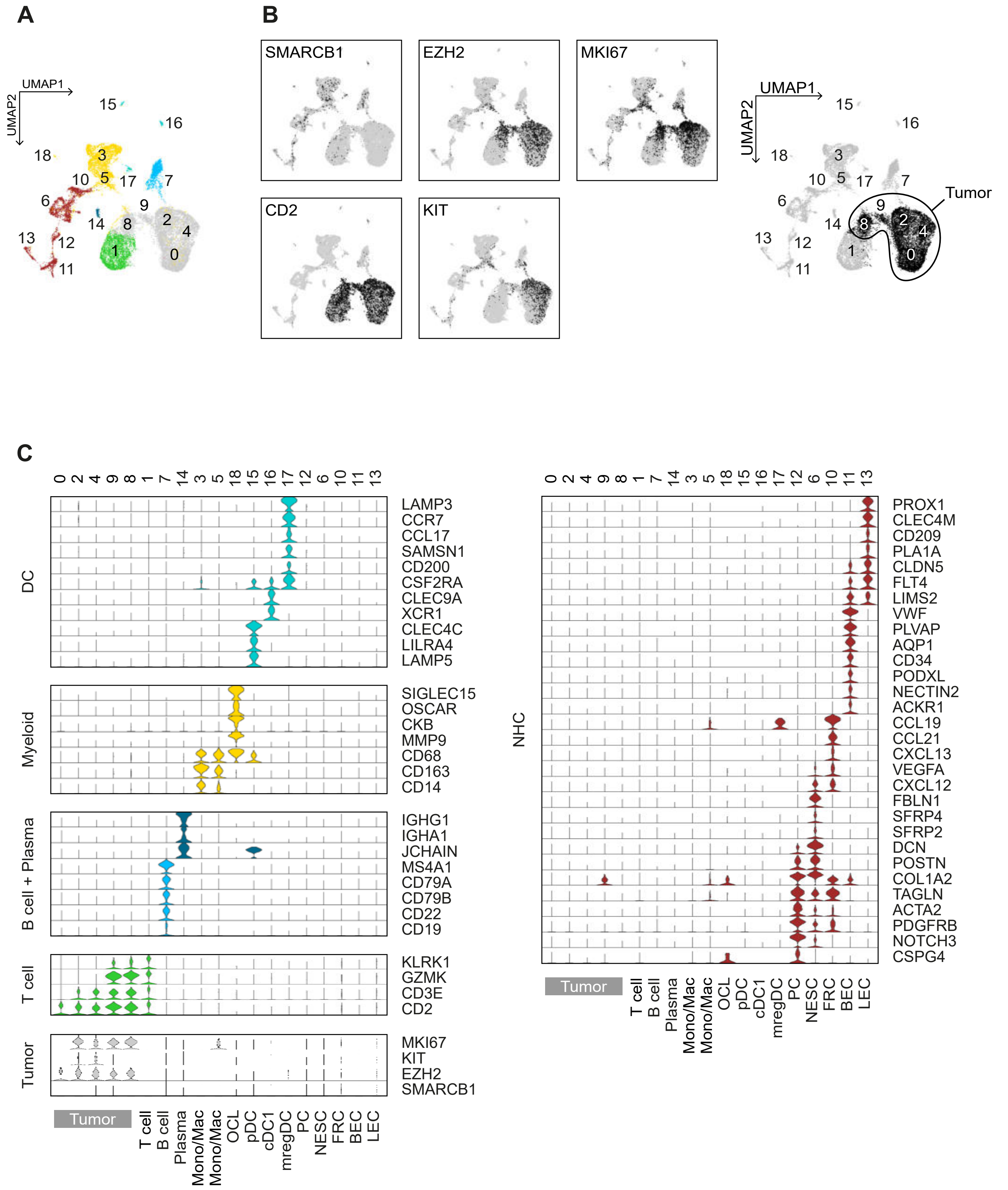


**Suppl. Figure 7. Detailed cell type annotation of human PTCL-NOS$^{SMARCB1-}$** (related to Figure 3).
(A) UMAP plot of the 19 clusters of the integrated human PTCL-NOS$^{SMARCB1-}$ scRNA-seq dataset.
(B) Differentiation of malignant versus non-malignant cells according to a 5-gene expression criterion.
(C) Violin plots classifying the different cell types within each major compartment.

## Supplementary Figure 8

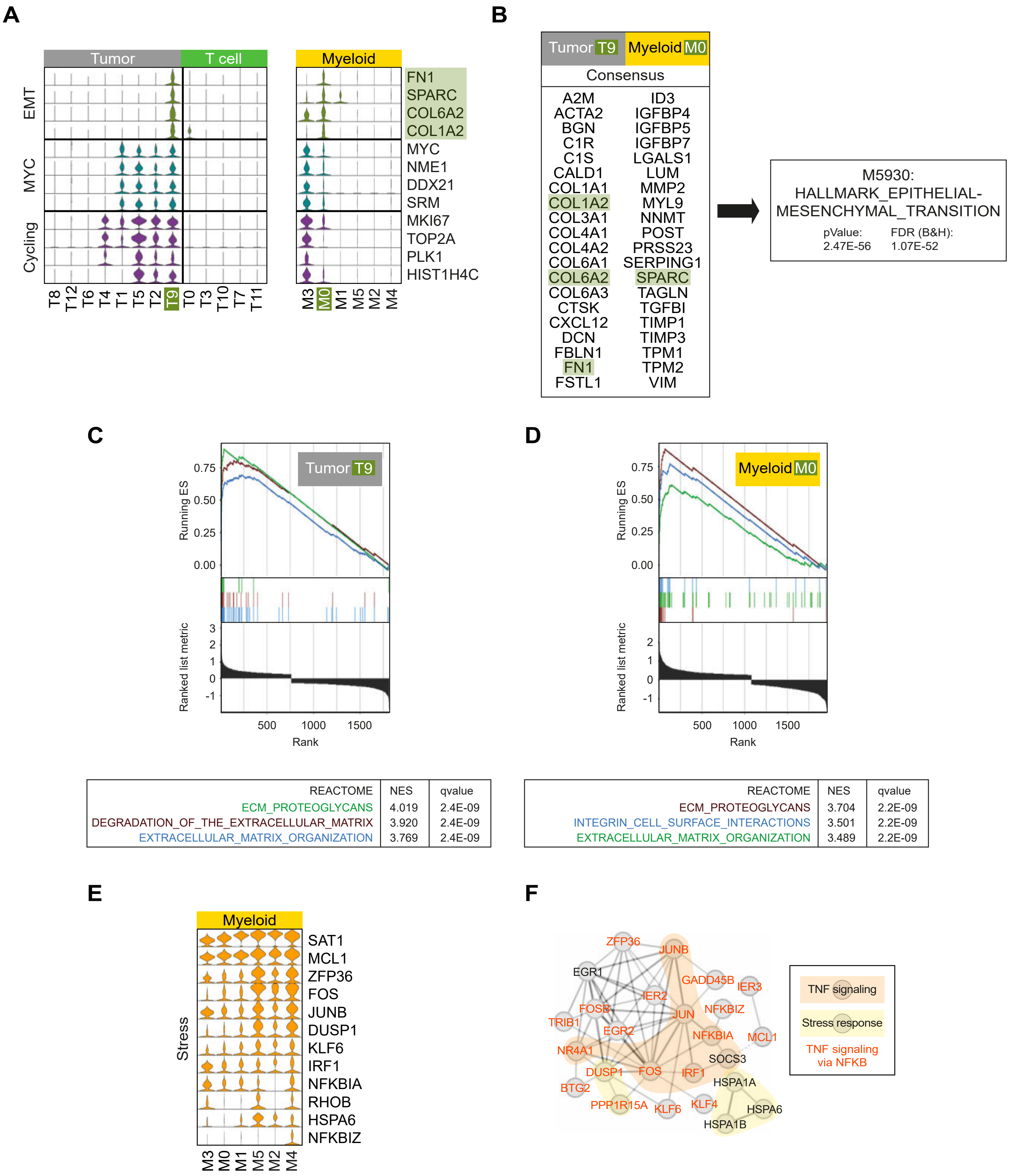


| REACTOME | NES | qvalue |
|---|---|---|
| ECM_PROTEOGLYCANS | 4.019 | 2.4E-09 |
| DEGRADATION_OF_THE_EXTRACELLULAR_MATRIX | 3.920 | 2.4E-09 |
| EXTRACELLULAR_MATRIX_ORGANIZATION | 3.769 | 2.4E-09 |

| REACTOME | NES | qvalue |
|---|---|---|
| ECM_PROTEOGLYCANS | 3.704 | 2.2E-09 |
| INTEGRIN_CELL_SURFACE_INTERACTIONS | 3.501 | 2.2E-09 |
| EXTRACELLULAR_MATRIX_ORGANIZATION | 3.489 | 2.2E-09 |

**Suppl. Figure 8. Distinct cancer hallmark metaprograms in human PTCL-NOS$^{SMARCB1-}$** (related to Figure 3).
(A) Violin plots revealing overlaps of Cycling, MYC and EMT programs in tumor cell and myeloid clusters. (B) A 30-gene consensus of the shared EMT program found in tumor cell cluster T9 and myeloid cluster M0. (C) Running enrichment scores (ES) of the T9_EMT program and (D) of the M0_EMT program, for each of the 3 top-ranked gene sets from the Molecular Signatures Database (MSigDB; https://gsea-msigdb.org; category C5_BP: ontology gene sets, biological process). (E) Violin plot of the stress program found in myeloid clusters. (F) Functional network analysis of the stress program using STRING database (www.string-db.org)[23]. Analysis settings: full STRING network; minimum required interaction score: medium confidence (0.400).

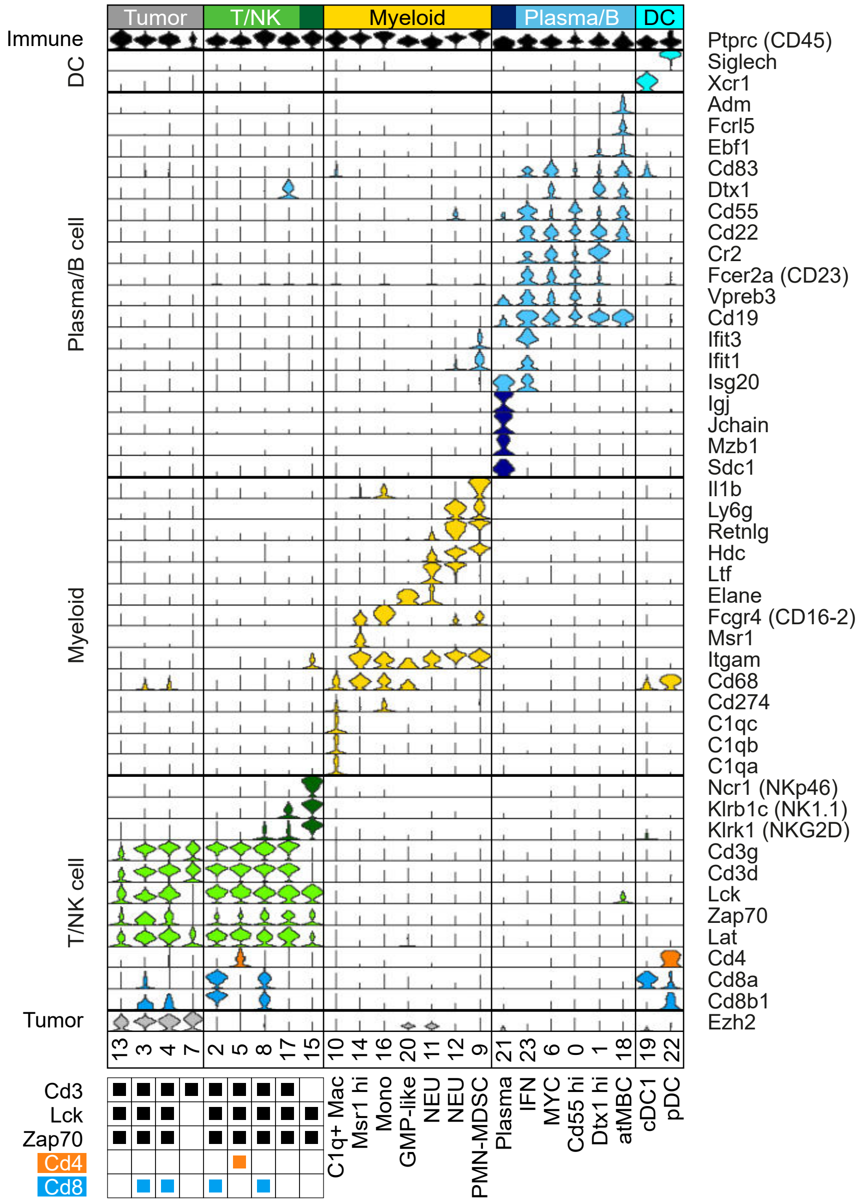


**Suppl. Figure 9. Detailed cell type annotation of murine WT and PTCL-NOS$^{Smarcb1-}$ samples** (related to Figure 5).
Violin plot showing the expression patterns of the specific marker genes used to classify different cell types of the integrated scRNA-seq dataset of murine WT and tumor spleens.

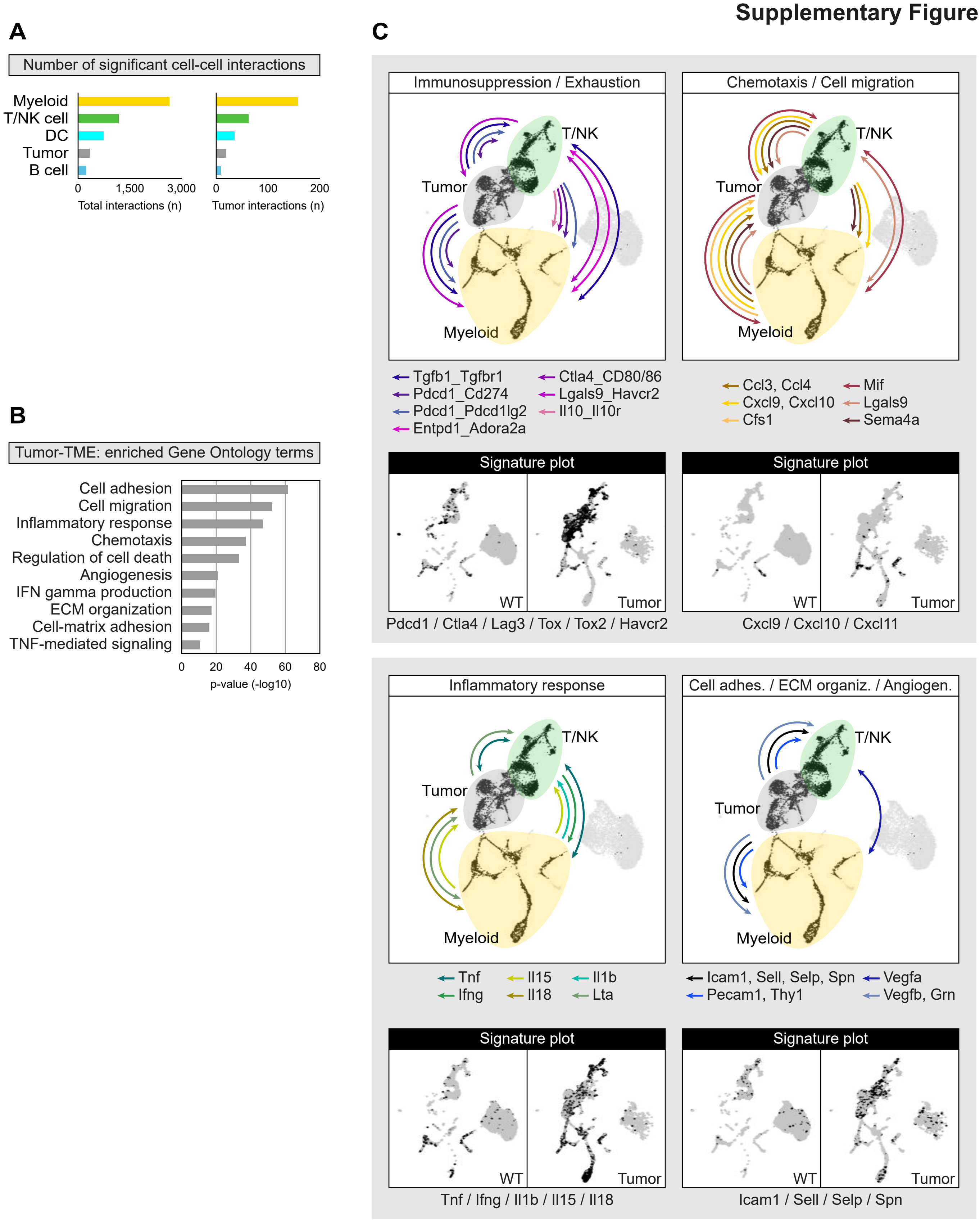


**Suppl. Figure 10. Cell-cell communication pathways in murine PTCL-NOS$^{Smarcb1-}$** (related to Figure 5).
(A) Bar graphs show the total number of significant cell-cell interactions (CCIs) between the different cellular compartments in the murine PTCL scRNA-seq data set (left), and the number of their interactions with tumor cell clusters (right). (B) Enriched GO terms of CCIs between tumor cell and TME clusters. (C) The upper panels show a schematic representation of the UMAP-projected communication between tumor cells and the myeloid and T/NK cell compartments and are based on the interactions predicted by CellPhoneDB, which were further analyzed using InterCellar and divided into the four functional groups Immunosuppression/Exhaustion, Chemotaxis/Cell migration, Inflammatory response and Cell adhesion/ECM organization/ Angiogenesis. Color-coded arrows indicate the direction of the respective L-R interaction, and bidirectional arrows indicate that both interacting cell types secrete the respective ligand. The lower panels show signature plots of selected factors as averaged mean expression of all signature genes in the different clusters, each separately in cells from WT (left) and tumor spleens (right). Source data of panel A are provided as a Source Data file.

# Supplementary Figure 11

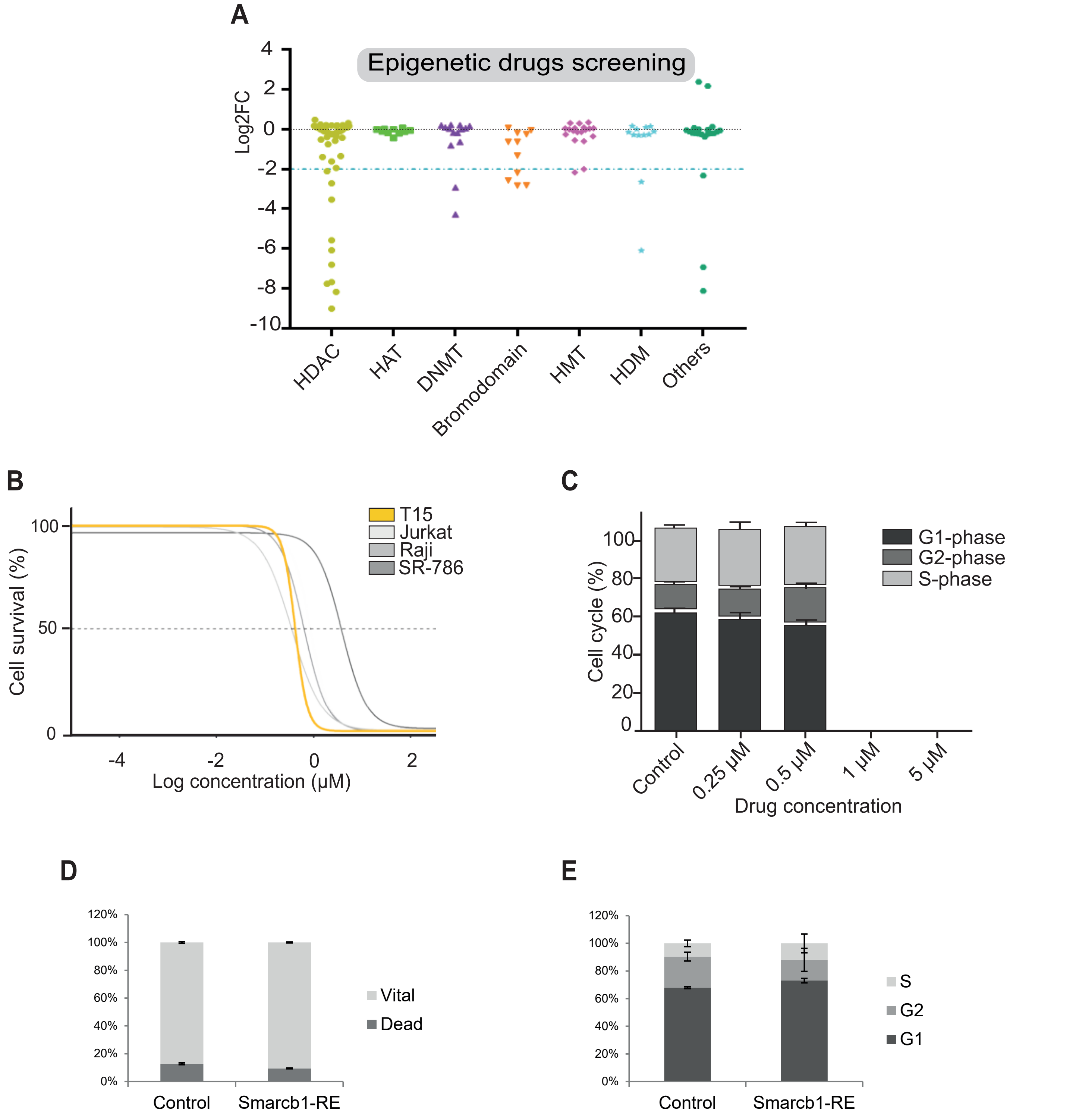


**Suppl. Figure 11. Effects of HDACi/SAHA treatment and Smarcb1 re-expression on T15 PTCL cells** (related to Figure 6). (A) Epigenetic drug screening (n = 140 drugs; see Supplementary Data S13) of murine Smarcb1-negative T15 cells and seven human NHL cell lines (in total n = 8). All compounds were screened in 1 μM and 10 μM concentration. Epigenetic drug classes are illustrated in different colors, each dot representing a different substance. Drug effect on T15 cells is represented as T15 vitality referred to NHL cell lines [$\log_2$ fold change (FC)]. (B) Survival curves showing the cell viability (%) of T15 cells and the three NHL cell lines Jurkat, Raji and SR-786 along a SAHA concentration range (0.1 nM to 100 μM, expressed in log units); n ≥ 4 biological replicates. (C) Titration of SAHA and the effect on cell cycle distribution of T15 cells (n = 3 biological replicates; data points for 1 and 5 μM are not shown due to massive cell death; all other data are presented as mean values +/- SEM). (D) Apoptosis assay and (E) cell cycle analysis of control and Smarcb1-reexpressing T15 cells using FACS (n ≥ 3; data are presented as mean values +/- SEM). Source data of panels A-E are provided as a Source Data file.

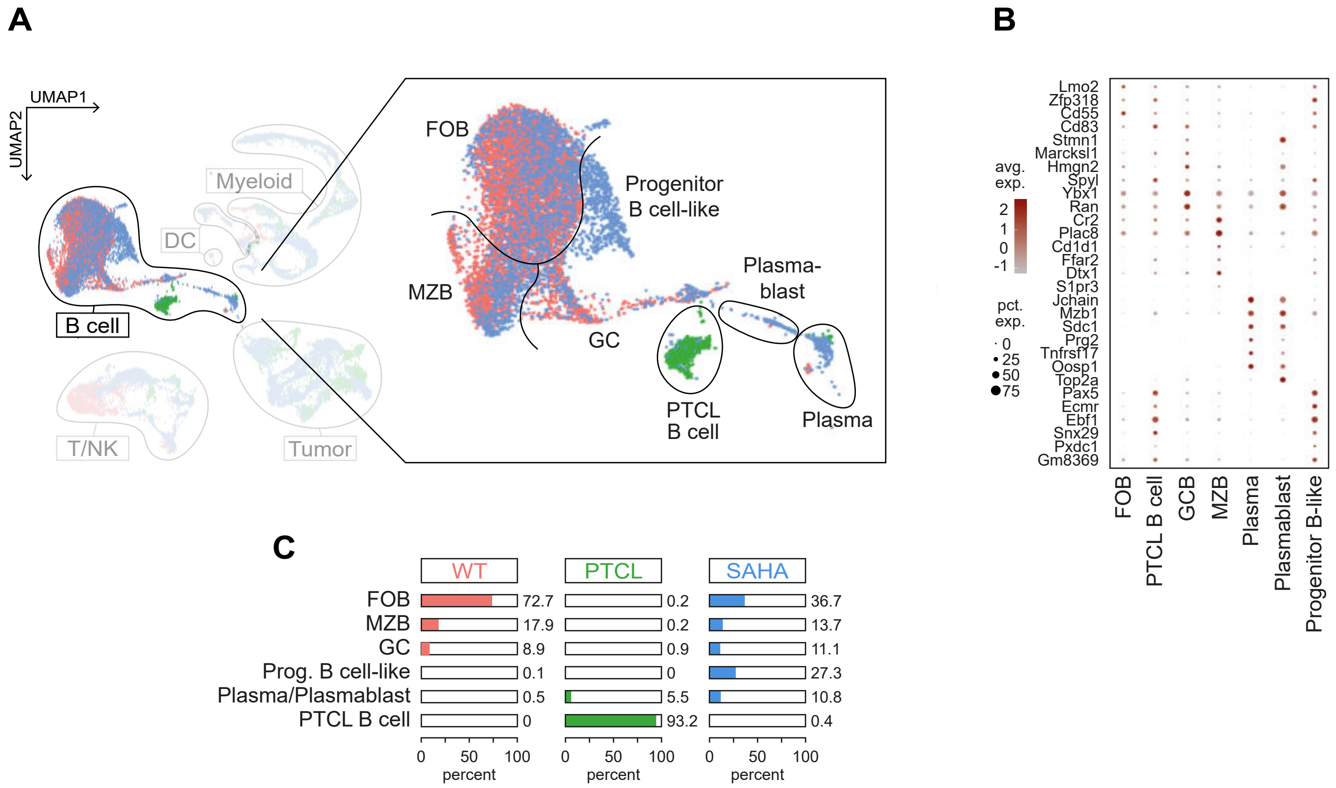


**Suppl. Figure 12. The B cell compartment of murine PTCL-NOS$^{Smarcb1-}$** (related to Figure 7). (A) Expanded view of the B cell compartment in the murine PTCL data set with a more detailed cell type annotation based on the dot plot shown in (B). (C) Quantitative analysis of the contributions of distinct B cell subtypes in the three sample groups. FOB, follicular B cell; GC, germinal center B cell; MZB, marginal zone B cell.

**Overview Supplementary Tables**


1 Structural variants affecting SMARCB1 in T-PLL patients and cell lines
2 MF clinical and histopathological features and SMARCB1 staining
3 MEITL and EATL cases show partial loss of SMARCB1 protein expression in immunohistochemical analysis
4 SMARCB1 protein expression correlates with age in PTCL-NOS patients
5 Mutational status of SMARCB1 in 4 SMARCB1-negative PTCL-NOS cases
6 Publicly available DNA methylation array data used in this study
7 Summary of animals used for tumor detection/model establishment and/or histology
8 Summary of animals used for T cell isolation and DNA methylation profiling
9 P values and FDR for GO term enrichment of differentially hyper- and hypomethylated CpGs
10 Quantitative information on the single-cell RNA sequencing of human tissue
11 Composition of cell culture media

**Table S1: Structural variants affecting SMARCB1 in T-PLL patients and cell lines.** The table lists breakpoint locations for gains and losses detected in T-PLL cases (n = 16) and the SUP-T11 cell line using the ACEseq tool and structural variants (SVs) detected by the Sophia algorithm.

| **ACEseq tool** | | | | | | | |
|---|---|---|---|---|---|---|---|
| PID | chr | start (hg19) | end (hg19) | length | TCN (total copy number) | | |
| case 121 | 22 | 16850007 | 29092560 | 12242554 | 12.513 | | |
| case 124 | 22 | 18949965 | 26842361 | 7892397 | 0.6987088 | | |
| **SOPHIA** | | | | | | | |
| sample | chr | start (hg19) | end (hg19) | chr | start (hg19) | end (hg19) | SV type |
| SUP-T11 | 16 | 62594925 | 62594926 | 22 | 23925814 | 23925815 | TRA |

**Table S2: MF clinical and histopathological features and SMARCB1 staining.** A total of 15 mycosis fungoides cases were stained for SMARCB1(INI1) protein expression. One case (case n. 2) displayed positive staining in most of the tumor cells (95%) with just a few scattered negative elements with irregular nuclear profiles located in the superficial dermis (5%). All the remaining MF cases that were tested showed indistinctively intense nuclear staining; thus, they were considered not to have the SMARCB1 mutation.

| Case | Age at diagnosis | Gender | Year of diagnosis | Diagnosis | SMARCB1 staining |
|---|---|---|---|---|---|
| **1** | 26 | F | 2011 | MF plaque stage | Positive |
| **2** | 33 | M | 2016 | MF early patch stage | Positive (5% negative) |
| **3** | 42 | M | 2016 | MF plaque stage | Positive |
| **4** | 22 | F | 2020 | MF plaque stage | Positive |
| **5** | 25 | M | 2022 | MF plaque stage | Positive |
| **6** | 78 | F | 2023 | MF plaque stage | Positive |
| **7** | 70 | M | 2023 | MF histological transformation | Positive |
| **8** | 46 | F | 2020 | MF tumor phase | Positive |
| **9** | 87 | M | 2020 | MF histological transformation | Positive |
| **10** | 74 | M | 2021 | MF tumor phase | Positive |
| **11** | 67 | F | 2023 | MF early patch stage | Positive |
| **12** | 63 | M | 2023 | MF, early patch stage | Positive |
| **13** | 65 | M | 2023 | MF early patch stage | Positive |
| **14** | 81 | F | 2023 | MF early patch stage | Positive |
| **15** | 87 | M | 2022 | MF plaque stage | Positive |

**Table S3: MEITL and EATL cases show partial loss of SMARCB1 protein expression in immunohistochemical analysis.** A total of 39 MEITL and 15 EATL cases were stained for SMARCB1(INI1) protein expression. While the majority of cases (n = 28 MEITL and n = 10 EATL) showed SMARCB1 expression in most tumor cells (~100%), a subset of cases (n = 11 MEITL and n = 5 EATL) showed a partial loss (≤ 70% of cells expressing SMARCB1).

| Subtype | n total | Partial loss |
|---|---|---|
| MEITL | 39 | 11 |
| EATL | 15 | 5 |

**Table S4: SMARCB1 protein expression correlates with age in PTCL-NOS patients.** List of patients included to study the correlation of SMARCB1 protein expression (assessed with IHC) and age of the patient. Patients were included from 3 different studies. [2]These cases belong to the extended cohort and were specifically selected for SMARCB1 protein loss.

| Study | Prot.exp | Age |
|---|---|---|
| TENOMIC | 1 | 73.7 |
| TENOMIC | 1 | 49.6 |
| TENOMIC | 1 | 69.3 |
| TENOMIC | 1 | 62.4 |
| TENOMIC | 1 | 72.7 |
| TENOMIC | 1 | 65 |
| TENOMIC | 1 | 23 |
| TENOMIC | 1 | 59.9 |
| TENOMIC | 1 | 61.8 |
| TENOMIC | 1 | 37.1 |
| TENOMIC | 1 | 45.3 |
| TENOMIC | 1 | 69.9 |
| TENOMIC | 1 | 71.9 |
| TENOMIC | 1 | 44.5 |
| TENOMIC | 1 | 74.9 |
| Tzankov, 2007 | 1 | 51 |
| Tzankov, 2007 | 1 | 63 |
| Tzankov, 2007 | 1 | 73 |
| Tzankov, 2007 | 0 | 76 |
| Tzankov, 2007 | 1 | 76 |
| Tzankov, 2007 | 1 | 70 |
| Tzankov, 2007 | 1 | 57 |
| Tzankov, 2007 | 1 | 63 |
| Tzankov, 2007 | 1 | 85 |
| Tzankov, 2007 | 1 | 37 |
| Tzankov, 2007 | 1 | 81 |
| Tzankov, 2007 | 1 | 77 |
| Tzankov, 2007 | 1 | 76 |
| Tzankov, 2007 | 1 | 69 |
| NHL-BFM (P1) | 0 | 8 |
| NHL-BFM (P2) | 0 | 8 |
| NHL-BFM (P3) | 0 | 7 |
| NHL-BFM (P4) | 0 | 12 |
| NHL-BFM[2] (P5) | 0 | 9 |
| Oslo1[2] (P6) | 0 | 23 |
| Oslo2[2] (P7) | 0 | 28 |
| MSKCC[2] (P8) | 0 | 24 |
| Vancouver[2] (P9) | 0 | 14 |
| NHL-BFM | 1 | 2 |
| NHL-BFM | 1 | 10 |
| NHL-BFM | 1 | 15 |
| NHL-BFM | 1 | 16 |
| NHL-BFM | 1 | 12 |
| NHL-BFM | 1 | 13 |
| NHL-BFM | 1 | 18 |
| NHL-BFM | 1 | 16 |

**Table S5: Mutational status of SMARCB1 in 4 SMARCB1-negative PTCL-NOS cases.** Results of SMARCB1 targeted NGS using the TruSight enrichment protocol. All identified variants in the SMARCB1 gene are listed and classified into pathogenic or benign according to Richards et al.[22] [1]Patient number 5 showed a homozygous deletion in the analyzed region (Suppl. Figure 4), [2]Patient number 8 showed a loss of exon 1 (Suppl. Figure 4).

| Case | Gen state | Variant | HGVS nomenclature | Classification |
|---|---|---|---|---|
| P1 | WT/Mut | c.157C>T | NM_003073.4:c.157C>T | Pathogenic |
| | | | p.Arg53* | |
| | Het del | | | |
| P2 | WT/WT | c.362+7C>T | NM_003073.4:c.362+7C>T | Benign |
| | | | p.? | |
| | | c.1119-41G>A | NM_003073.4:c.1119-41G>A | Benign |
| | | | p.? | |
| P3 | WT/WT | | | |
| P4 | WT/LOH | | | |
| P5[1] | Hom del | | | |
| P6 | Mut/LOH | c.644G>A | NM_001317946.2:c.644G>A | Pathogenic |
| | | | p.Trp215* | |
| P7 | Hom del | | | |
| P8[2] | Mut | Exon1 loss | | |
| P9 | WT/Mut | c.555_556dupGC | NM_003073.5:c.555_556dupGC | |
| | | | p.Leu186fs*24 | |

**Table S6: Publicly available DNA methylation array data used in this study.**

| Sample Type | Number | Publication | PubmedID |
|---|---|---|---|
| HSCs/precursors | 6 | Lee, 2012 | 23074194 |
| Macrophages | 12 | Garcia-Gomez, 2017 | 28973458 |
| | | Vento-Tormo, 2016 | 26758199 |
| Monocytes | 8 | Kennedy, 2018 | 29914364 |
| CD34, ISP, CD4, CD8, TCRn, TCRp | 12 | Touzart, 2021 | 34039737 |
| RespCD4, CD8Treg, GammaDelta | 20 | Bergmann, 2019 | 30337361 |
| CD3 | 5 | Hassler, 2016 | 27705804 |
| PTCL | 4 | This study | - |
| T-LBL | 48 | Tian, 2020 | 32234760 |
| ALCL | 10 | Hassler, 2016 | 27705804 |
| gdTCL/HSTL | 15 | Bergmann, 2019 | 30337361 |

**Table S7: Summary of animals used for tumor detection/model establishment and/or histology**

| Mouse model | Mouse ID | Experimental group | Sex | Age [weeks] |
|---|---|---|---|---|
| CD4-cre::Smarcb1 $^{fl/fl}$ | 4 | PTCL | F | 9 |
| CD4-cre::Smarcb1 $^{fl/fl}$ | 5 | PTCL | M | 11 |
| CD4-cre::Smarcb1 $^{fl/fl}$ | 9 | PTCL | M | 12 |
| CD4-cre::Smarcb1 $^{fl/fl}$ | 10 | PTCL | M | 11 |
| CD4-cre::Smarcb1 $^{fl/fl}$ | 12 | PTCL | M | 11 |
| CD4-cre::Smarcb1 $^{fl/+}$ | 16 | Control | M | 9 |
| CD4-cre::Smarcb1 $^{fl/fl}$ | 20 | PTCL | M | 12 |
| CD4-cre::Smarcb1 $^{fl/fl}$ | 23 | PTCL | F | 11 |
| CD4-cre::Smarcb1 $^{fl/fl}$ | 24 | PTCL | F | 10 |
| CD4-cre::Smarcb1 $^{fl/+}$ | 27 | Control | M | 11 |
| CD4-cre::Smarcb1 $^{fl/fl}$ | 29 | PTCL | F | 11 |
| CD4-cre::Smarcb1 $^{fl/fl}$ | 30 | PTCL | F | 10 |
| CD4-cre::Smarcb1 $^{fl/+}$ | 36 | Control | M | 10 |
| CD4-cre::Smarcb1 $^{fl/+}$ | 37 | Control | F | 8 |
| CD4-cre::Smarcb1 $^{fl/+}$ | 38 | PTCL | F | 8 |
| CD4-cre::Smarcb1 $^{fl/fl}$ | 40 | Control | F | 8 |
| CD4-cre::Smarcb1 $^{fl/fl}$ | 47 | SAHA | M | 10 |
| CD4-cre::Smarcb1 $^{fl/fl}$ | 49 | SAHA | M | 10 |
| CD4-cre::Smarcb1 $^{fl/fl}$ | 54 | SAHA | M | 10 |
| CD4-cre | 142 | Control | M | 22 |

**Table S8: Summary of animals used for T cell isolation and DNA methylation profiling**

| Mouse model | Mouse ID | Experimental group | Sex | Age [weeks] |
|---|---|---|---|---|
| Smarcb1 $^{fl/fl}$ | 1346 | Control | F | 13 |
| Smarcb1 $^{fl/fl}$ | 1347 | Control | F | 14 |
| Smarcb1 $^{fl/fl}$ | 1348 | Control | M | 14 |
| Smarcb1 $^{fl/fl}$ | 1349 | Control | M | 14 |
| Smarcb1 $^{fl/fl}$ | 1362 | Control | M | 13 |
| CD4-cre::Smarcb1 $^{fl/fl}$ | 9 | PTCL | M | 12 |
| CD4-cre::Smarcb1 $^{fl/fl}$ | 10 | PTCL | M | 11 |
| CD4-cre::Smarcb1 $^{fl/fl}$ | 20 | PTCL | M | 12 |
| CD4-cre::Smarcb1 $^{fl/fl}$ | 23 | PTCL | F | 11 |
| CD4-cre::Smarcb1 $^{fl/fl}$ | 24 | PTCL | F | 10 |

**Table S9: P values and FDR for GO term enrichment of differentially hyper- and hypomethylated CpGs.**

| GO_term | pVal | Group | FDR |
|---|---|---|---|
| regulation of myeloid cell apoptotic process | 0.000017809 | Hyper | 0.048482 |
| myeloid cell apoptotic process | 0.000032569 | Hyper | 0.056535 |
| positive T cell selection | 0.000043791 | Hyper | 0.056535 |
| lymphocyte differentiation | 0.0000037344 | Hyper | 0.033748 |
| mononuclear cell differentiation | 0.0000078353 | Hyper | 0.035404 |
| | | | |
| myeloid leukocyte differentiation | 0.000088637 | Hypo | 0.10013 |
| cellular response to peptide | 0.000072150 | Hypo | 0.093145 |
| enzyme-linked receptor protein signaling pathway | 0.00016206 | Hypo | 0.14646 |
| cellular response to oxygen-containing compound | 0.000038691 | Hypo | 0.087412 |
| negative regulation of cell communication | 0.000018501 | Hypo | 0.083599 |

**Table S10: Quantitative information on the single-cell RNA sequencing of human tissue**

| CaseTable S4 | Sex | Age(years) | Cells | Median reads per cell | Median genes per cell | Genes targeted | Genes detected | Number of reads from cells called from this sample | Reads Mapped Confidently to Probe Set (%) |
|---|---|---|---|---|---|---|---|---|---|
| 2 | F | 8 | 2,108 | 3,579 | 1,071 | 18,082 | 15,657 | 12,454,769 | 98.76 |
| 3 | M | 7 | 6,026 | 1,243 | 441 | 18,082 | 15,979 | 10,142,036 | 98.76% |
| 1 | F | 8 | 4,991 | 3,543 | 1,093 | 18,082 | 17,902 | 28,606,447 | 98.92% |
| 4 | F | 12 | 3,872 | 1,994 | 709 | 18,082 | 17,865 | 9,923,980 | 98.74% |
| 5 | M | 9 | 4,128 | 3,389 | 1,281 | 18,082 | 16,817 | 26,709,275 | 98.98% |

**Table S11: Composition of cell culture media.** PTCL-NOS: Peripheral T-cell lymphoma, T-ALL: T-cell acute lymphoblastic leukemia, ALCL: Anaplastic large-cell lymphoma, BL: Burkitt's lymphoma, HL: histiocytic lymphoma.

| Cell line | Medium | Supplements (vol/vol) | Manufacturers |
|---|---|---|---|
| T15 (Smarcb1 neg. PTCL-NOS) | RPMI-1640 | 10% FBS | Merck, #S0115 |
| | | 1% HEPES (1 M) | Merck, #L1613 |
| | | 1% penicillin/streptomycin (10,000 U/ml) | Gibco, #15140122 |
| | | 1% sodium pyruvat (100 mM) | Gibco, #11360070 |
| | | 1% L-glutamin (200 mM) | Gibco, #25030081 |
| | | 0.1% -β-mercaptoethanol (50 nM) | Gibco, #31350010 |
| Jurkat (T-ALL) | RPMI-1640 | 10% FBS | Merck, #S0115 |
| Karpas (ALCL) | | 1% penicillin/streptomycin (10,000 U/ml) | Merck, #L1613 |
| SR-786 (ALCL) | | | |
| SU-DHL-1 (ALCL) | | | |
| Raji (BL) | | | |
| Daudi (BL) | | | |
| U-937 (HL) | | | |
