# Supplementary material for: Lack of SMARCB1 expression characterizes a subset of human and murine peripheral T-cell lymphomas
Source: Nat Commun. 2024 Oct 3;15:8571. doi: 10.1038/s41467-024-52826-0 (PMC11452211; doi:10.1038/s41467-024-52826-0)
Supplement: Supplementary file 3 — Description of Additional Supplementary Files [file 41467_2024_52826_MOESM3_ESM.pdf]

Description of Additional Supplementary Files

Supplementary Data 1: Concordantly hypermethylated genes in both species. Supervised analysis (t-test: σ/σmax > 0.4, q < 0.01) of SMARCB1-negative PTCLs and CD3-positive T cells in mouse and human samples was performed using the OMICS Explorer 3.6. Murine hyper- and hypomethylated genes were lift-overed to human genes using Biomart and then overlapped with resulting genes from the same comparison in human samples.

Supplementary Data 2: Concordantly hypermethylated genes in both species - stringent cutoff. Supervised analysis (t-test: σ/σmax > 0.4, q < 1e-5) of SMARCB1-negative PTCLs and CD3-positive T cells in mouse and human samples was performed using the OMICS Explorer 3.6. Murine hyper- and hypomethylated genes were lift-overed to human genes using Biomart and then overlapped with resulting genes from the same comparison in human samples.

Supplementary Data 3: Differentially expressed genes of human SMARCB1-negative PTCL samples (cf. Suppl. Figure 7A). For details on generation of DEGs see: https://satijalab.org/seurat/. p_val: unadjusted p-value, avg_log2FC: log fold-change of the average expression between the two groups (cluster x vs. rest), pct.1: percentage of cells where the feature (gene) is detected in the first group, pct.2: percentage of cells where the feature (gene) is detected in the second group, p_val_adj: adjusted p-value, based on Bonferroni correction using all features (genes) in the dataset.

Supplementary Data 4: Comparison of Tumor/T-cell clusters (T0-T12) with cancer hallmark metaprograms (MP1-41): numbers

Supplementary Data 5: Comparison of Tumor/T-cell clusters (T0-T12) with cancer hallmark metaprograms (MP1-41): genes

Supplementary Data 6: Comparison of Myeloid clusters (M0-M6) with cancer hallmark metaprograms (MP1-41): numbers

Supplementary Data 7: Comparison of Myeloid clusters (M0-M6) with cancer hallmark metaprograms (MP1-41): genes

Supplementary Data 8: Quantitative information on the single-cell RNA sequencing of mouse tissue. In house samples were processed with CellRanger v3.0.2 (10X Genomics) and reads were aligned to the mouse reference genome (mm10). The table shows output values obtained for each of the four in house samples when running CellRanger “count” with default parameters.

Supplementary Data 9: Differentially expressed genes of integrated murine WT/PTCL samples (cf. Figure 5A). For details on generation of DEGs see: https://satijalab.org/seurat/. p_val: unadjusted p-value, avg_log2FC: log fold-change of the average expression between the two groups (cluster x vs. rest), pct.1: percentage of cells where the feature (gene) is detected in the first group, pct.2: percentage of cells where the feature (gene) is detected in the second group, p_val_adj: adjusted p-value, based on Bonferroni correction using all features (genes) in the dataset.

Supplementary Data 10: Comparison of mouse scRNA-seq clusters (C0-C23) with cancer hallmark metaprograms (MP1-41): numbers

Supplementary Data 11: Comparison of mouse scRNA-seq clusters (C0-C23) with cancer hallmark metaprograms (MP1-41): genes

Supplementary Data 12: InterCellar (Interlandi et al., 2021) output after cell-cell interactions analysis of PTCL scRNA-seq data.

Supplementary Data 13: Epigenetics screening library (Cayman Chemicals)

Supplementary Data 14: Differentially expressed genes of integrated murine WT/PTCL/SAHA samples (cf. Figure 7A). For details on generation of DEGs see: https://satijalab.org/seurat/. p_val: unadjusted p-value, avg_log2FC: log fold-change of the average expression between the two groups (cluster x vs. rest), pct.1: percentage of cells where the feature (gene) is detected in the first group, pct.2: percentage of cells where the feature (gene) is detected in the second group, p_val_adj: adjusted p-value, based on Bonferroni correction using all features (genes) in the dataset.
