# Supplementary material for: Lack of SMARCB1 expression characterizes a subset of human and murine peripheral T-cell lymphomas
Source: Nat Commun. 2024 Oct 3;15:8571. doi: 10.1038/s41467-024-52826-0 (PMC11452211; doi:10.1038/s41467-024-52826-0)
Supplement: Supplementary file 5 — Reporting Summary [file 41467_2024_52826_MOESM5_ESM.pdf]

nature portfolio

Corresponding author(s): Dr. med. Kornelius Kerl

Last updated by author(s): Sep 18, 2024

Reporting Summary

Nature Portfolio wishes to improve the reproducibility of the work that we publish. This form provides structure for consistency and transparency in reporting. For further information on Nature Portfolio policies, see our Editorial Policies and the Editorial Policy Checklist.

Statistics

For all statistical analyses, confirm that the following items are present in the figure legend, table legend, main text, or Methods section.

n/a	Confirmed	
☐	☒	The exact sample size (*n*) for each experimental group/condition, given as a discrete number and unit of measurement
☐	☒	A statement on whether measurements were taken from distinct samples or whether the same sample was measured repeatedly
☐	☒	The statistical test(s) used AND whether they are one- or two-sided<br>*Only common tests should be described solely by name; describe more complex techniques in the Methods section.*
☒	☐	A description of all covariates tested
☐	☒	A description of any assumptions or corrections, such as tests of normality and adjustment for multiple comparisons
☐	☒	A full description of the statistical parameters including central tendency (e.g. means) or other basic estimates (e.g. regression coefficient) AND variation (e.g. standard deviation) or associated estimates of uncertainty (e.g. confidence intervals)
☐	☒	For null hypothesis testing, the test statistic (e.g. *F*, *t*, *r*) with confidence intervals, effect sizes, degrees of freedom and *P* value noted<br>*Give P values as exact values whenever suitable.*
☒	☐	For Bayesian analysis, information on the choice of priors and Markov chain Monte Carlo settings
☒	☐	For hierarchical and complex designs, identification of the appropriate level for tests and full reporting of outcomes
☒	☐	Estimates of effect sizes (e.g. Cohen's *d*, Pearson's *r*), indicating how they were calculated

Our web collection on statistics for biologists contains articles on many of the points above.

Software and code

Policy information about availability of computer code

Data collection

SMARCB1 gene expression levels of T-cell lymphoma and T-PLL samples were mined from the TENOMIC database (LYSA consortium).
DNA methylation profiles of primary human PTCL (n = 4) were generated using the Infinium MethylationEPIC BeadChip (Illumina). For comparison, we mined publicly available data of different T-cell subpopulations generated with the Infinium HumanMethylation450 BeadChip (Illumina).
DNA methylation profiles of the PTCL-NOS Smarcb1- mice (n = 5) and corresponding control groups including non-neoplastic samples isolated from the spleen (n = 5) and splenic Cd3+ sorted cells (n = 5) were generated using a custom service for Infinium Mouse Methylation BeadChip.

Human scRNA-seq
Isolation of cells for Chromium Fixed RNA profiling was done according to protocol CG000632_RevB (10X Genomics). Briefly, after deparaffiniz¬ation and rehydration the tissue sections were dissociated using a heated gentleMACS Octo dissociator for FFPE tissue (Miltenyi Biotec) with freshly prepared Dissociation Enzyme Mix containing Liberase TH (Millipore Sigma). After sample filtration, single-nuclei suspensions were counted using Invitrogen Countess 3 Automated Cell Counter (Thermo Fisher Scientific). To generate fixed RNA gene expression libraries, we used the Chromium Next GEM Single Cell Fixed RNA Sample Preparation Kit, 16 rxn (PN-1000414). Single-nuclei suspensions were mixed with human transcriptome probes and hybridized for 20 h at 42°C and then further processed according to protocol CG000527_RevE for multiplexed libraries (10X Genomics). One million cells per sample were used for probe hybridization. GEMs were prepared with a targeted recovery of 80,000 cells in each 16-plex library.

Murine scRNA-seq
For single-cell preparation, murine spleens were collected independently of gender and minced using scalpels. Enzymatic (StemPro™ Accutase™ (Gibco™, #A1110501); 37°C) and mechanical dissociation were applied for 20 min. Afterwards, cells were washed with PBS. Erythrocytes were lysed using ACK lysing buffer (Gibco™, #A1049201) according to the manufacturer's protocol. Then, to remove non-viable

cells, they were stained with 7-AAD (eBioscience™, #00-6993-50) and sorted (BD FACSAria-II). Manual cell counting of sorted cells was performed using Trypan blue staining. The single-cell suspension was processed further using Chromium Single Cell 3’ Gel Bead Kit v2 (10x Genomics) according to the manufacturer’s protocol.

RNA isolation and bulk sequencing of murine T15 cell line /Smarcb1 reexpression: 3 groups: control, Smarcb1+ and Smarcb1 neg (each in triplicate). RNA was isolated from tumor tissue using RNeasy Mini Kit (QIAGEN, #74104). RNA sequencing was carried out by the Core Facility of the University Hospital Muenster, Germany. For this, the ultra II RNA directional library prep Kit for Illumina (New England Biolabs, #E7760S) was used and samples were sequenced using the Next-Seq 2000 sequencing System (1X72 Cycles, 25 Mio single reads/sample) at the Genomics Core Facility (University Hospital Münster, Münster).

Data analysis

DNA sequencing and copy number analysis of the SMARCB1 gene
For targeted next-generation sequencing of SMARCB1 exons and flanking intronic sequences, the TruSight DNA target enrichment was used for library preparation and sequencing was performed on the MiSeq platform (Illumina Inc., San Diego, CA, USA). The generated fastq files were analyzed by SeqPilot software version 5.1.0 (Module SeqNext, JSI Medical Systems) for alignment and variant calling (hg19). For copy number determination, DNA was hybridized to an OncoScan CNV assay (Thermo Fisher Scientific, Waltham, MA, USA). Analysis was performed using the Chromosome Analysis Suite Software version 4.0 (Thermo Fisher Scientific, Waltham, MA, USA). Only copy number alterations larger than 50kb, encompassing at least 20 informative probes with a median log2ratio of >0.2 or <-0.2 and copy number neutral losses of heterozygosity larger than 5 Mb were considered for further analyses. Additionally, FISH for the SMARCB1 locus was performed as described previously.

DNA methylation analysis using Illumina Infinium arrays
Raw idat files generated from the human samples were normalized using the preprocess Illumina function without background correction from the minfi package within the R statistical program (www.R-project.org, version 4.1.2). We converted human samples run on EPIC arrays into virtual 450K arrays in order to cross-compare all studies. For the mouse samples the raw idat files were normalized with GenomeStudio (v2011.1; methylation module 1.9.0; Illumina Inc., San Diego, CA, USA) applying default settings and internal normalization controls. Subsequently, beta values were calculated representing the percentage of DNA methylation at a certain cytosine base. For downstream analysis, loci with a detection p value > 0.01, rs loci and loci on gonosomes were excluded from further analysis. Differentially methylated loci for human and mouse samples were identified using the OMICS Explorer 3.6 (Qlucore; Lund, Sweden).
In order to identify meaningful hyper- or hypomethylated biological processes or molecular functions a gene ontology enrichment analysis was performed using WEB-based Gene Set Analysis Toolkit. Heatmaps, UMAPs (Uniform Manifold Approximation and Projection) and boxplots were generated in R using the pheatmap, umap and ggplot2 package, respectively.

Murine und human tumor samples single-cell RNA sequencing:
Note: * indicates settings/specifications for mouse scRNA-seq samples; ** for human Fixed RNA samples.
Briefly, we used the R package Seurat to perform initial quality control and filtering, integration, dimensionality reduction, clustering and differentially expressed gene (DEG) analysis. The raw Illumina bcl files were demultiplexed using Cell Ranger (*v.3.0.2 and **v.7.1.0) ‘mkfastq’ step with default specifications. Individual sample gene expression matrices were generated using the Cell Ranger *count and Cell Ranger **multi pipeline using genome version *mm10 and **GRCh38-2020-A provided by 10X Genomics Cell Ranger. Data analysis was performed using R (*v.3.6.1 and **v.4.3.1) and Seurat (both v.3.1.3 and v.4.3.0).

For initial quality control by Seurat, genes that were expressed in fewer than three cells and cells that expressed fewer than *50 genes and **200 genes were excluded from analysis. Briefly, each dataset was filtered on dataset-specific parameters for genes per cell, UMIs per cell and percentage of mitochondrial genes. We performed normalization using the Seurat function NormalizeData (method = “LogNormalize”, scale.factor = 10,000). Finally, highly variable genes (n = 2,000) were calculated with the selection method “vst”.

Bulk sequencing of murine T15 cell line : Raw FASTQ files were obtained and, after explorative quality control with FastQC5 and MultiQC6, Salmon7 was used for pseudo-alignment and quantification of the samples to the mouse transcriptome (downloaded from Ensembl, release 94). Default parameters were used. Further analyses were performed in R. We employed the Bioconductor package tximport8 to summarize transcript-level estimates computed by Salmon for a gene-level analysis. To find differentially expressed genes, we used the package DESeq29 and tested for SMARCB1 re-expression versus control conditions. Only genes with adjusted p-value < 0.05 were considered (Benjamini-Hochberg correction).

For manuscripts utilizing custom algorithms or software that are central to the research but not yet described in published literature, software must be made available to editors and reviewers. We strongly encourage code deposition in a community repository (e.g. GitHub). See the Nature Portfolio guidelines for submitting code & software for further information.

Data

Policy information about availability of data

All manuscripts must include a data availability statement. This statement should provide the following information, where applicable:

- Accession codes, unique identifiers, or web links for publicly available datasets
- A description of any restrictions on data availability
- For clinical datasets or third party data, please ensure that the statement adheres to our policy

Bulk-RNA sequencing data from murine T15 cell line /Smarcb1 reexpression samples, single-cell RNA-seq from human and murine tumors and human/murine DNA methylation data have been deposited in NCBI's Gene Expression Omnibus and raw data is accessible under accession numbers GSE190273 for bulk RNA-seq data of T15 cell lines, GSE190274 for mouse PTCL single cell RNA-seq, GSE249566 for human and murine PTCL DNA methylation array data and GSE254299 for human PTCL single-nuclei RNA-seq data. No source code or custom scripts were developed in this study. Data analysis was performed using publicly available packages. Details are available upon request. We used publicly available scRNA-seq data of mouse control spleens from the Tabula Muris Consortium available at figshare (https://figshare.com/articles/dataset/Single-cell_RNA-seq_data_from_microfluidic_emulsion_v2_/5968960).

Research involving human participants, their data, or biological material

Policy information about studies with human participants or human data. See also policy information about sex, gender (identity/presentation), and sexual orientation and race, ethnicity and racism.

Reporting on sex and gender	Male and female samples were utilized equally. As this study describes a very rare disease, we included all patients and did not select for age or sex/gender in advance. Sex/gender of patients was determined based on self-report.
Reporting on race, ethnicity, or other socially relevant groupings	As we investigated an extremely rare phenotype, we included all patients that met the inclusion criteria of a SMARCB1 negative T cell lymphoma. Race, ethnicity or other groupings were not considered.
Population characteristics	Not applicable.
Recruitment	Experiments were performed on archived patient material. No patients were recruited specifically for this study
Ethics oversight	The SMARCB1 expression analysis in T-PLL has been approved by the Institutional Ethical Review Board of the Medical Faculty of Ulm University (21/16 and 463/19 (02.13. 2020), in PTCLs from the TENOMIC Consortium Biobank by the Comité de Protection des Personnes Ile de France 08-009, in MEITL/EATLs by the Commission nationale d'éthique de la recherche sur l'être humain (CER-VD, protocol 382/14). CAYA PTCL-NOS patients were registered into the NHL-BFM study center database after informed consent of the legal guardians had been obtained (Ethikkommission der Ärztekammer Westfalen-Lippe und der Westfälischen Wilhelms Universität; file number: 2017-077-f-S).

Note that full information on the approval of the study protocol must also be provided in the manuscript.

Field-specific reporting

Please select the one below that is the best fit for your research. If you are not sure, read the appropriate sections before making your selection.

☒ Life sciences ☐ Behavioural & social sciences ☐ Ecological, evolutionary & environmental sciences

For a reference copy of the document with all sections, see nature.com/documents/nr-reporting-summary-flat.pdf

Life sciences study design

All studies must disclose on these points even when the disclosure is negative.

Sample size	For patient samples, no predetermination on samples size was possible as we investigated an extremely rare phenotype. For mouse or cell line experiments, no statistical methods were used to predetermine sample sizes. Sample sizes were determined to be adequate based on statistical testing of magnitude and consistency between groups in the experimental phase and were in line with previous publications.
Data exclusions	No samples were excluded from the study.
Replication	For single-cell transcriptome analyses of murine spleens/ tumors 2 biological samples were considered.<br>For in vitro experiments at least 3 technical triplicates were considered (if not otherwise stated). All attempts at replication were successful.<br>Depending on the method used, several cell lines were analyzed (up to seven NHL cell lines).
Randomization	For in vivo experiments, after tumor engraftment mice were randomized into two different groups (vehicle and treated mice).
Blinding	No blinding was performed in the data analyses. Patient samples were genetically characterized but not compared to a control group, so blinding was not relevant to this study.

Reporting for specific materials, systems and methods

We require information from authors about some types of materials, experimental systems and methods used in many studies. Here, indicate whether each material, system or method listed is relevant to your study. If you are not sure if a list item applies to your research, read the appropriate section before selecting a response.

Materials & experimental systems

n/a	Involved in the study	
☐	☒	Antibodies
☐	☒	Eukaryotic cell lines
☒	☐	Palaeontology and archaeology
☐	☒	Animals and other organisms
☒	☐	Clinical data
☒	☐	Dual use research of concern
☒	☐	Plants

Methods

n/a	Involved in the study	
☒	☐	ChIP-seq
☒	☐	Flow cytometry
☒	☐	MRI-based neuroimaging

Antibodies

Antibodies used	anti-SMARCBl (Clone 25/BAF47, BD Bioscience, cat# 612110 and cat# ab16645) for IHC and Western Blot of human tissue.<br>FITC anti-mouse CD3ε Antibody Cat. # 100306 for FACS isolation of CD3 positive murine spleen cells.<br>For multiplexed immunofluorescence analysis, spleen slices were stained in the MACSima imaging system using antibodies against B220 (RA3-6B2, Miltenyi Biotec, APC, 1:50), Ly6G (1A8, Miltenyi Biotec, PE, 1:50) and EZH2 (REA907, Miltenyi Biotec, APC, 1:50).
Validation	All antibodies used are commercially available and were validated by the manufacturer.

Eukaryotic cell lines

Policy information about cell lines and Sex and Gender in Research

Cell line source(s)	Human cell lines: Jurkat (T-ALL), Karpas (ALCL), SR-786 (ALCL), SU-DHL-1 (ALCL), Raji (BL), Daudi (BL), U-937 (HL). Gift from Prof. Claudia Rössig, University Children's Hospital Münster, Münster, Germany. Additionally, the human T-cell leukemia cell line SUP-T11 (DSMZ, #ACC605) was analyzed.<br>Murine cell line: T15 (Smarcb1 neg. PTCL-NOS). Gift from Charles W. M. Roberts, Dana-Farber Cancer Institute, BOS, USA
Authentication	Cell lines were authenticated by STR profiling through the Institute for Forensic Medicine (University of Münster) or the Institute of Human Genetics (University Medical Center Ulm).
Mycoplasma contamination	All cell lines were tested in the beginning of any experiment and in between (every ~8weeks) using mycoplasma PCR in house. We confirm that all cell lines were tested negative for mycoplasma.
Commonly misidentified lines<br>(See ICLAC register)	The study did not involve misidentified lines.

Animals and other research organisms

Policy information about studies involving animals; ARRIVE guidelines recommended for reporting animal research, and Sex and Gender in Research

Laboratory animals	Cd4-cre (kindly provided by Dr. Maren Lindner, UKM) and Smarcb1fl/fl mice were obtained from the Jackson Laboratory (https://www.jax.org/).
Wild animals	The study did not involve wild animals.
Reporting on sex	Male and female mice were utilized equally.
Field-collected samples	The study did not involve field-collected samples.
Ethics oversight	Protocols and animal housing were in accordance with all guidelines provided by the local regulatory authorities (reference number TVA-84-02.04.2018.A296; Government of NRW, Germany).

Note that full information on the approval of the study protocol must also be provided in the manuscript.
